# Development and preliminary psychometric evaluation of the hemodialysis patient care dependency scale (HD-CDS)

**DOI:** 10.1371/journal.pone.0358118

**Published:** 2026-09-15

**Authors:** Cong Liang, Hongyan Niu, Lina Xue

**Affiliations:** 1 Blood Purification Center, The First People’s Hospital of Changzhou, Changzhou, Jiangsu, China; 2 School of Medicine, Jiangsu University, Zhenjiang, Jiangsu, China; Phramongkutklao College of Medicine, THAILAND

## Abstract

**Background:**

Assessing the care dependency of patients undergoing hemodialysis (HD) may be crucial for disease management and control across various stages. Currently, the tools used to measure care dependency are primarily applicable to stroke patients, and comprehensive and reliable assessment tools specifically for patients undergoing HD are lacking. In this study, we developed a care dependency scale suitable for patients undergoing HD and evaluated its psychometric properties.

**Methods:**

This exploratory, sequential mixed-methods study comprised two stages: (1) scale development and (2) psychometric evaluation, including refining the scale and assessing its psychometric properties. The items were generated through a literature review, semi-structured interviews, the nominal group technique, the Delphi method, and pilot testing. We recruited 395 patients undergoing HD for the psychometric evaluation. The psychometric properties of the HD-CDS were examined through item analysis, exploratory factor analysis, assessments of content and convergent validity, internal consistency, and test–retest reliability; 39 clinically stable participants were included in the test–retest analysis.

**Results:**

The final scale comprises 17 items across three dimensions (50 scoring criteria in total): basic needs, HD treatment, and social support. The content validity index was 0.914. The exploratory factor analysis accounted for 69.683% of the total item variance, and all items demonstrated good discriminative ability. Convergent validity was supported by a strong negative correlation with the Barthel Index (*r* = −0.715, p < 0.001). The overall scale showed good internal consistency reliability (Cronbach’s α = 0.893, split-half reliability = 0.885), and test–retest reliability among the 39 participants included in the analysis was excellent (intraclass correlation coefficient = 0.965). Significant associations were found between the HD patient care dependency scale scores and education, dialysis sessions, vascular access type, dialysis vintage, and pre‑dialysis blood pressure (all *p* < 0.01).

**Conclusions:**

The HD-CDS demonstrated promising preliminary psychometric properties in this single-center sample and may provide a useful framework for assessing care dependency among patients undergoing HD. Further multicenter studies, including independent structural validation and inter-rater reliability assessment, are required before routine clinical implementation.

## Background

According to the China Kidney Disease Network report, the incidence of uremia requiring renal replacement therapy in China is 122.19 per million population, with a prevalence of 442.13 per million population [1]. Hemodialysis (HD) is a form of renal replacement therapy for patients with acute and chronic renal failure [2]. Although treatment can prolong the lives of patients with end-stage renal disease, it cannot fully replace kidney function. Patients undergoing HD often face issues such as reduced exercise tolerance [3], poor nutritional status [4], and calcium-phosphorus metabolism disorders [5], leading to a decline in physical function. Furthermore, the exacerbation of comorbidities such as cardiovascular disease [6] and cognitive decline [7] can further impact patients’ quality of life, potentially necessitating long-term care and medication treatment. These effects impose an economic burden on patients and contribute to social pressure.

Nursing dependency primarily refers to situations in which patients must rely on professional caregivers due to reduced self-care abilities or increased care needs [8]. Rather than referring to an objective state, nursing dependency emphasizes a subjective experience involving five fundamental concepts: functional limitations, needs, self-care deficits, seeking support, and unmet needs. This perspective highlights the impact of individual differences on the demand for nursing services.

Patients undergoing HD exhibit unique characteristics across the disease spectrum, which is reflected in their specific nursing needs. These patients rely on caregivers long-term for vascular access assessment and maintenance [9], as well as education on medication knowledge [10], guidance on dietary and fluid intake [11], instructions for functional exercise [12], and psychological counseling. Thus, these patients demonstrate a high level of dependency on HD nurses. Additionally, patients often travel between hospitals and their residences during treatment intervals, during which changes in their condition may occur while they are not able to be monitored by healthcare professionals. Thus, comprehensively assessing the degree of nursing dependency in patients undergoing HD is important for revealing patients’ specific needs, the healthcare issues they face, and nurses’ workloads. Nursing assessment is a critical component of the nursing process [13], as such assessments provide an important reference for meeting patients’ health needs and helping them enhance their self-care abilities, as well as optimizing nursing resource allocation. Selecting appropriate measurement tools for patient assessment is of particular importance.

Existing assessment tools for care dependency in patients undergoing HD remain inadequate, particularly for evaluating HD-related complications [14] and vascular access maintenance needs. This shortcoming prevents the comprehensive and effective assessment of the care dependency levels for patients undergoing HD. We operationally define care dependency in patients undergoing HD as the need for professional nursing assistance due to reduced self‑care ability or increased care needs, distinct from frailty (physiological vulnerability) and functional status (actual performance of daily activities). Based on Henderson’s theory, we hypothesized three core dimensions: basic physiological needs, needs related to the dialysis procedure itself, and needs for social and informational support. This study aimed to develop the HD-CDS and conduct a preliminary evaluation of its psychometric properties in patients undergoing HD.

## Methods

### Design

This exploratory, sequential mixed-methods study was conducted from August 2024 to December 2024. The research comprised two stages: (1) scale development (August 1, 2024, to September 30, 2024) and (2) psychometric evaluation (October 1, 2024, to December 1, 2024), which included refining the scale and assessing its psychometric properties.

### Phase 1: Scale development

#### Creating the item pool.

The scale’s initial item pool was developed through (1) a comprehensive literature review and (2) semi-structured interviews.

We conducted a search using keywords such as “hemodialysis,” “blood purification,” “end-stage renal disease,” “dependency,” “care needs,” “care dependency,” “self-care capability,” “assessment tools,” and “scales.” The databases searched included CNKI, Wanfang Database, VIP Journal Full-text Database, China Biomedical Literature Database, American Guidelines Network, PubMed, Web of Science, and Cochrane. The search aimed to obtain content related to the care dependency of patients undergoing HD. Additionally, existing comprehensive care dependency assessment tools and specialized care dependency assessment tools were referenced.

We developed a semi-structured interview outline to optimize the item pool, and conducted face-to-face interviews. The primary objectives of the interviews were to (1) understand the reasons for care dependency in patients undergoing HD from the perspective of HD medical staff, thereby enhancing the scientific rigor and reliability of item setting; and (2) to streamline the initial items to avoid overly lengthy expert consultation questionnaires, thereby ensuring a certain degree of expert engagement and effectiveness. The semi-structured interviews employed purposive sampling and concluded when no new themes emerged during data processing. The interview participants, which included doctors and nurses, were selected from the HD center of a tertiary hospital in Changzhou, Jiangsu Province. The inclusion criteria were as follows: (1) at least 5 years of work experience in the field of blood purification; (2) a professional technical title of intermediate level or above and an educational background of at least a bachelor’s degree; (3) familiarity with HD and relevant professional knowledge regarding patients undergoing HD; (4) a high level of enthusiasm for this research, with the ability to communicate promptly; and (5) the provision of informed consent for the interview. The qualitative content analysis method was applied to analyze the review data.

#### Preliminarily evaluating the items.

We used the nominal group technique (NGT), Delphi expert consultation, and a pilot test to preliminarily evaluate and revise the items.

The NGT was used to evaluate and revise the initial item pool and dimensions by structuring face-to-face meetings with 10 experts to facilitate discussion and reach a consensus. The research team comprised one nursing mentor, two physicians from the blood purification center, the head nurse, six specialized nurses from the blood purification center, and one nursing graduate student. The team members’ expertise spanned various fields, including nursing management, blood purification nursing, nephrology, and public health. The NGT comprised 5 stages: introducing and explaining the purpose and procedure of the meeting, the silent generation of ideas, the presentation of ideas in a round robin, the clarification of any unclear ideas/items, and participant voting on the importance of ideas [15].

The project and dimensions were revised, and the scale was improved through two rounds of Delphi expert consultations. We invited 18 experts from Jiangsu, Jiangxi, and Sichuan provinces in China. The inclusion criteria for experts were as follows: (1) at least 10 years of work experience in relevant fields, including nursing management, nursing research, and medical statistics; (2) a professional title of associate senior or above and an educational background of at least a bachelor’s degree; (3) familiarity with the professional knowledge related to HD and patients undergoing HD; (4) a willingness to participate in this study; and (5) a high level of enthusiasm during the consultation process, with timely feedback on results. The exclusion criterion was non-response within 2 weeks of the start of each consultation round. Experts were contacted via electronic or paper mail, and the results of the expert consultation were evaluated based on the level of expert engagement, authority, and the degree of consensus among expert opinions. The positive coefficient of experts was assessed by the response rate [16]. An authority coefficient (Cr) of over 0.8 is considered a high expert authority coefficient [17]. For the coordination coefficient of experts, Kendall’s coefficients of concordance (Kendall’s W) of 0.5 or above is considered a high correlation, and *p* < 0.05 is considered to indicate significance [18]. The coefficient of variation (CV) is an important basis for index deletion. Items meeting the following conditions were retained: mean ≥ 3.5 and CV < 0.25. The experts’ input was also incorporated to modify the items and formulate a draft scale.

### Phase 2: Psychometric evaluation

In this stage, the original version of the HD patient care dependency scale (HD-CDS) was used to evaluate the nursing dependency of patients undergoing HD. We employed item analysis, validity tests, and reliability assessments to filter the items and evaluate the psychometric properties.

#### Item analysis.

Item analysis was conducted to determine whether each item in the original instrument should be retained or deleted. Low-quality items were removed from the scale. Two methods were used for item analysis. (1) The critical ratio method represents the difference values of the extreme group comparisons. Based on the total score, the nursing care dependency scores for patients undergoing HD were ranked, and groups were formed from the top 27% and the bottom 27% to create high- and low-scoring groups. An independent-samples *t* test was then used to determine whether there were significant differences in the average values of the different item measurements. If the difference value was not significant, we considered deleting that item. (2) The correlation coefficient method calculates the correlation between each item and the total score. Items with a correlation coefficient below 0.4 were considered for deletion.

#### Validity analysis.

**Content validity:** The content validity of the original scale was assessed. Six experts participated in the consultation. The inclusion criteria for the experts were the same as those for the Delphi portion. The experts reviewed the wording, comprehensiveness, and relevance of each item using a 4-point scale [19]. Each expert rated each item from 1 (not relevant) to 4 (very relevant and concise). The item-level content validity index (I-CVI) was calculated as the number of experts who rated an item as 3 or 4, divided by the total number of experts. The scale-level content validity index (S-CVI) is the mean of the I-CVIs of all items. The I-CVI should be greater than or equal to 0.78, and the S-CVI should be greater than or equal to 0.8 [20].

**Construct validity:** The three‑factor structure was first hypothesized based on Henderson’s theory and qualitative findings, then tested by exploratory factor analysis (EFA) in Statistical Package for Social Sciences (SPSS) version 27.0.1 to assess structural validity. Principal component analysis was used for factor extraction, followed by orthogonal Varimax rotation. The scale was evaluated by 32 trained nurses using paper-based forms, strictly adhering to the three-level item criteria. Ambiguous items were scored conservatively (i.e., the higher score was selected) to prioritize patient safety. Data suitability was confirmed by Bartlett’s sphericity test (*p* < 0.001) and Kaiser–Meyer–Olkin (KMO) measure (minimum threshold = 0.7) [21].

Factors with eigenvalues > 1 were retained [21,22], determined through scree plot analysis. Varimax rotation (orthogonal method) enhanced interpretability [23], with factors named per item loadings [24]. A factor loading of ≥ 0.40 was selected a priori as a practical threshold for interpreting salient loadings [25].

#### Convergent validity.

Convergent validity refers to the extent to which scores on an instrument are associated with measures of theoretically related constructs [26]. Because no established gold-standard instrument is available for assessing care dependency specifically in patients undergoing HD, convergent validity was examined using Pearson’s correlation. The Barthel Index [27] was selected as the comparator because it assesses functional independence in activities of daily living, a construct conceptually related to care dependency. However, it does not directly assess hemodialysis-specific care needs. A negative correlation was hypothesized because higher HD-CDS scores indicate greater care dependency, whereas higher Barthel Index scores indicate greater functional independence.

#### Reliability analysis.

The Cronbach’s α coefficient and the split-half Spearman–Brown coefficient were used to assess the internal consistency and reliability of the scale and dimensions. The test–retest reliability was assessed using intraclass correlation coefficients (ICCs) and Pearson’s r to determine the scale’s stability. ICCs were assessed using a two‑way mixed model with agreement.

### Participants

All patients met the early screening, diagnostic, and prevention guidelines for chronic kidney disease (2022 edition) [28] and underwent HD. The inclusion criteria were as follows: (1) patients aged 18 years or older and (2) patients who voluntarily accepted follow-up by the investigators. Patients who exhibited uncooperative behavior during the survey or were undergoing both HD and peritoneal dialysis were excluded. This study was conducted at the Blood Purification Center of Changzhou First People’s Hospital. Patient recruitment and data collection spanned from October 1, 2024, to December 1, 2024. Patients undergoing HD participated in the pilot test (n = 32), and psychological measurement assessments were conducted. The sample size was calculated based on 5–10 times the number of items [29], taking into consideration a 10% invalid sample rate, resulting in a total of 50 × (5–10) × (1 + 10%) cases; thus, the ideal range for the on-site survey sample size was determined to be between 275–550 cases. Considering logistical factors such as study duration, human resource requirements, and budget constraints, the final sample size ultimately included 395 cases. Forty participants underwent the two-week retest. One participant was excluded because of a clinically important change in health status during the retest interval, resulting in a final test–retest analysis sample of 39 participants.

### Data collection

Data were collected throughout the entire study. Appropriate locations and dates were selected for the semi-structured interviews, which were audio-recorded. Each participating healthcare professional underwent one to two sessions, with an average duration of 20–30 min per session. In the pilot study, we documented participating nurses’ comments on the items. The HD-CDS was completed by primary nurses who had been trained on the scale content. These nurses evaluated patients who met the inclusion and exclusion criteria, and the assessment continued until all items of the scale were fully completed. During the psychological testing phase, we developed training strategies based on the comments from 32 nurses who participated in the pilot study before conducting the scale assessments. Each assessment took approximately 12–15 min to complete. The researchers explained the study’s purpose and the method for administering the scale assessments. The participating nurses administered the assessments independently or with the researchers’ assistance. Forty participants completed a second HD-CDS assessment two weeks after the initial assessment. During data verification, one participant was found to have experienced an acute gastrointestinal bleeding event during the retest interval. Because this represented a clinically important change in health status and violated the stability assumption underlying test–retest reliability assessment, this participant was excluded. Therefore, 39 participants were included in the test–retest analysis.

### Statistical analysis

The data were analyzed using SPSS for Mac version 27.0.1. Descriptive statistics were used to describe the participants’ demographic characteristics, including counts, frequencies (%), means, and standard deviations.

In the item analysis, if the corrected item-total correlation was below 0.30 and Cronbach’s α increased when the item was deleted, the item was removed from the scale [30]. Additionally, items with *p* values > 0.05 in the critical ratio method were considered to lack discriminative ability and were deleted.

For exploratory factor analysis, principal component analysis was used for factor extraction, followed by orthogonal Varimax rotation. Components with eigenvalues ≥ 1.00 were retained, and a cumulative explained variance of ≥ 60% was considered satisfactory [31].

In the reliability analysis, Cronbach’s α and test–retest reliability coefficients were calculated from the total (395) and retest (39) samples. The prespecified thresholds for acceptable internal consistency were > 0.80 for both Cronbach’s α and the split-half coefficient [32]. An ICC value between 0.70 and 1.00 was considered to indicate excellent stability, 0.60 to 0.70 good stability, 0.40 to 0.60 reasonable stability, and < 0.40 poor stability [33]. A Pearson’s *r* > 0.30 indicated good stability [34].

Parametric or nonparametric tests were applied based on data distribution to examine the associations between sociodemographic/clinical variables and HD‑CDS total scores. The data were approximately normally distributed; thus, parametric tests were used for most analyses. Nonparametric alternatives were applied when the normality assumption was violated. All tests were two‑tailed, and a *p* value < 0.05 was considered to indicate significance.

### Ethical considerations

This study was approved by the Ethics Committee of the First Hospital of Changzhou City, Jiangsu Province (ethics approval number: 2024-099). Written informed consent was obtained from all participants. The researchers informed participants of the study purpose and procedures before data collection. Participation was voluntary, and participants were assured that they could withdraw from the study without providing a reason. Data were kept confidential and used solely for research purposes.

## Results

### Scale development

We reviewed concept analyses, literature reviews, and measurement research on nursing dependence to understand the concept and its structure. Based on these reviews, we conceptualized nursing dependence in patients undergoing HD as a state in which patients depend on HD nurses for professional assistance due to decreased self-care ability or increased nursing needs. Through the literature review process, we ultimately referenced Henderson’s Theory of Basic Human Needs [35], the INICIARE instrument [36], and the Kidney Disease Outcomes Quality Initiative guidelines from the National Kidney Foundation [37]. After incorporating the semi-structured interview findings and drawing from the experiences of all research team members, we established scoring criteria based on the difficulties encountered in the HD nursing process. Consequently, we produced 4 primary items (dimensions), 13 secondary items, and 47 tertiary items (scoring criteria).

After one round of NGT, 4 secondary items and 20 tertiary items were added. As a result, the scale comprised 4 primary items, 17 secondary items, and 60 tertiary items (scoring criteria). Specific initial items are presented in S1 File.

There were 18 experts who participated in two rounds of Delphi consultations. In the first round, 18 questionnaires were collected, yielding a 100% response rate; in the second round, 17 were collected, yielding a 94.44% response rate. A total of 13 experts (72.22%) and 4 experts (23.53%) provided suggestions in the first and second rounds, respectively. The high questionnaire and suggestion return rates indicate a high level of engagement among the experts. However, the decrease in the number of experts providing feedback in the second round compared with the first suggests a convergence of expert opinions. The authority coefficients of the experts in the first and second rounds were 0.88 and 0.90, respectively. The coefficient (Cr) was determined through the experts’ familiarity with the items (Cs) and the basis for their judgments (Ca), calculated as Cr = (Cs + Ca)/ 2. The Kendall’s W coefficients for the first round of expert consultations were 0.158 (*p* < 0.05), 0.196 (*p* < 0.05), and 0.689 (*p* < 0.05) for the primary, secondary, and tertiary items, respectively. In the second round, the coefficients were 0.235 (*p* < 0.05), 0.148 (*p* < 0.05), and 0.290 (*p* < 0.05). Although significant (all *p* < 0.05), the relatively low Kendall’s W values for the first‑ and second-level items (ranging from 0.148 to 0.235) indicate only fair to moderate agreement among experts. The third-level items showed higher agreement (0.689 in round 1 and 0.290 in round 2). In the first round of expert consultations, based on expert suggestions, ‘3. Healthcare’ and ‘4. Social Support’ were merged into ‘3. Social Support,’ and ‘3.1 Health Promotion’ was revised to ‘Decision Support.’ Additionally, 11 third-level items with importance scores < 3.5 and/or coefficients of variation > 0.25 were deleted based on their importance ratings and variability.

The order of entries besides the first-level entries was adjusted based on their importance ratings, and entries were modified or added according to the experts’ suggestions and comments. Specific deletions, order adjustments, modifications, and the rationale for these changes are provided in S1 File. In the second round of expert consultation, all entries received an importance rating of ≥ 3.5, with a coefficient of variation of ≤ 0.24. The experts’ comments and suggestions still focused on the third-level entries, with specific modifications and the rationale for these changes detailed in S1 File. Finally, we created a scale for the pilot test, including 3 primary items, 17 secondary items, and 50 tertiary items (scoring criteria).

In the pilot test, 32 nurses each evaluated one patient undergoing HD using a scale assessment. We gained an in-depth understanding of each nurse’s assessment methods and integrated the strengths from various approaches. Ultimately, we standardized the scale assessment methods to include information system reviews, patient conversations, and physical examinations. Specific items were aligned with the most appropriate assessment methods. Furthermore, we established an additional rule: when a patient meets two scoring criteria for a secondary item simultaneously, the higher score will be selected for evaluation to better ensure the patient’s safety during care.

### Psychometric evaluation

#### General characteristics of participants.

This study included 395 patients undergoing maintenance HD (62.0% male, mean age 57.8 ± 13.3 years). Most received thrice-weekly HD (71.1%), with autogenous fistulas being the predominant vascular access (81.3%). The left forearm was the most common access site (71.1%). Participants had a mean dialysis vintage of 65.5 ± 65.7 months and a mean ultrafiltration rate of 4.13% ± 1.19%. Pre-dialysis blood pressure averaged 138.3/82.0 mmHg. Educational backgrounds varied, ranging from primary education (25.1%) to college-level (27.3%). Detailed characteristics are presented in Table 1.

**Table 1:** Demographic characteristics of the study participants.

Variables	Total sample (N = 395) n (%)
**Sex**	
Female	150 (38.0)
Male	245 (62.0)
**HD Sessions per Week**	
1 session	5 (1.3)
2 sessions	29 (7.3)
3 sessions	281 (71.1)
4 sessions	30 (7.6)
5 sessions	8 (2.0)
Irregular sessions	42 (10.6)
**Vascular Access Type**	
Temporary Catheter	30 (7.6)
Arteriovenous Graft	8 (2.0)
Autogenous Fistula	321 (81.3)
Tunneled Catheter	36 (9.1)
**Vascular Access Site**	
Right Forearm	44 (11.1)
Right Upper Arm	1 (0.3)
Right Femoral Vein	26 (6.6)
Right Jugular Vein	34 (8.6)
Left Forearm	281 (71.1)
Left Upper Arm	3 (0.8)
Left Femoral Vein	4 (1.0)
Left Jugular Vein	2 (0.5)
**Education**	
Primary or below	99 (25.1)
Junior High School	66 (16.7)
High School/Vocational	81 (20.5)
College	108 (27.3)
Bachelor’s or above	41 (10.4)
	**Mean ± SD**
**Age (Years)**	57.8 ± 13.3
**Dialysis Vintage (Months)**	65.5 ± 65.7
**Ultrafiltration Rate (%)**	4.1 ± 1.2
**Pre-HD SBP (mmHg)**	138.3 ± 28.9
**Pre-HD DBP (mmHg)**	82.0 ± 14.2

HD: hemodialysis; SD: standard deviation; SBP: systolic blood pressure; DBP: diastolic blood pressure.

#### Item analysis.

**Critical ratio method:** In this study, the three-level items of the scale serve as scoring criteria, with each criterion corresponding to a fixed score. Thus, only the first- and second-level items were analyzed using the critical ratio method. The patients were divided into two groups according to their total scores, with the top 27% and the bottom 27% compared using independent-samples *t* tests. All first- and second-level items had *p* values < 0.05, demonstrating significance. This finding suggests that all first- and second-level items exhibit good discriminative ability, as detailed in Table 2.

**Table 2:** Critical ratio item analysis (mean ± SD; N = 395).

Items	Mean		*t*	*p value*
	Low-Scoring Group (n = 144)	High-Scoring Group (n = 129)		
Item 1	2.24 ± 0.51	8.04 ± 6.30	10.422	*<* 0.001
Item 1.1	0.01 ± 0.08	0.98 ± 1.41	7.839	*<* 0.001
Item 1.2	1.01 ± 0.17	1.81 ± 0.98	9.022	*<* 0.001
Item 1.3	0.20 ± 0.40	1.78 ± 1.57	11.081	*<* 0.001
Item 1.4	0.00 ± 0.00	0.91 ± 1.18	8.809	*<* 0.001
Item 1.5	1.01 ± 0.29	1.82 ± 1.20	7.482	*<* 0.001
Item 1.6	0.01 ± 0.08	0.74 ± 0.86	9.575	*<* 0.001
Item 2	8.51 ± 0.68	13.98 ± 4.14	14.819	*<* 0.001
Item 2.1	1.16 ± 0.37	1.91 ± 0.79	9.925	*<* 0.001
Item 2.2	1.02 ± 0.14	1.77 ± 0.63	13.131	*<* 0.001
Item 2.3	1.00 ± 0.00	1.60 ± 0.63	10.736	*<* 0.001
Item 2.4	1.00 ± 0.00	1.50 ± 0.50	11.402	*<* 0.001
Item 2.5	1.17 ± 0.41	2.09 ± 0.87	11.046	*<* 0.001
Item 2.6	1.08 ± 0.29	1.75 ± 0.73	9.841	*<* 0.001
Item 2.7	1.08 ± 0.34	1.95 ± 0.94	9.863	*<* 0.001
Item 2.8	1.01 ± 0.08	1.40 ± 0.49	9.022	*<* 0.001
Item 3	1.40 ± 0.62	3.97 ± 1.64	16.748	*<* 0.001
Item 3.1	1.20 ± 0.40	1.88 ± 0.61	10.807	*<* 0.001
Item 3.2	0.18 ± 0.42	1.54 ± 0.87	16.229	*<* 0.001
Item 3.3	0.02 ± 0.14	0.54 ± 0.52	11.118	*<* 0.001

**Correlation coefficient method:** Similarly, only the first- and second-level items were analyzed using the correlation coefficient method. The correlation coefficients for the first- and second-level items ranged from 0.313 to 0.954, all of which were positive. Item 3.3 (social support – material support) showed a corrected item-total correlation of 0.313 (Table 3). Despite this relatively low correlation, the item was retained for two reasons. First, qualitative findings from the NGT and expert panel discussions indicated that nurses considered this item clinically important: when a patient’s family is unable to provide adequate material or financial support, the patient’s care dependency increases, requiring the nurse to compensate for the unmet material support. Second, the item’s total score range is only 0–1 points; thus, its influence on the overall scale variance is limited, and its correlation with the total score remained significant (*p* < 0.001). Thus, Item 3.3 was retained due to its clinical relevance and significance. For details, see Table 3.

**Table 3:** Correlation-based item analysis (N = 395).

Items	CR	*p* value (CR)	Correlation with the total scale score	*p* value
Item 1	10.422**	*<* 0.001	0.954**	*<* 0.001
Item 1.1	7.839**	*<* 0.001	0.923**	*<* 0.001
Item 1.2	9.022**	*<* 0.001	0.759**	*<* 0.001
Item 1.3	11.081**	*<* 0.001	0.844**	*<* 0.001
Item 1.4	8.809**	*<* 0.001	0.779**	*<* 0.001
Item 1.5	7.482**	*<* 0.001	0.843**	*<* 0.001
Item 1.6	9.575**	*<* 0.001	0.765**	*<* 0.001
Item 2	14.819**	*<* 0.001	0.923**	*<* 0.001
Item 2.1	9.925**	*<* 0.001	0.662**	*<* 0.001
Item 2.2	13.131**	*<* 0.001	0.742**	*<* 0.001
Item 2.3	10.736**	*<* 0.001	0.814**	*<* 0.001
Item 2.4	11.402**	*<* 0.001	0.813**	*<* 0.001
Item 2.5	11.046**	*<* 0.001	0.739**	*<* 0.001
Item 2.6	9.841**	*<* 0.001	0.638**	*<* 0.001
Item 2.7	9.863**	*<* 0.001	0.538**	*<* 0.001
Item 2.8	9.022**	*<* 0.001	0.859**	*<* 0.001
Item 3	16.748**	*<* 0.001	0.616**	*<* 0.001
Item 3.1	10.807**	*<* 0.001	0.499**	*<* 0.001
Item 3.2	16.229**	*<* 0.001	0.635**	*<* 0.001
Item 3.3	11.118**	*<* 0.001	0.313**	*<* 0.001

*p < 0.05,**p < 0.01.

#### Content validity (CVI).

The expert group assessed the scale’s content validity. The I-CVI ranged from 0.833 to 1.000, thus exceeding the prespecified threshold of 0.78. The S-CVI was 0.914, exceeding the 0.8 threshold, indicating good content validity.

#### Exploratory factor analysis.

**Fig 1:**
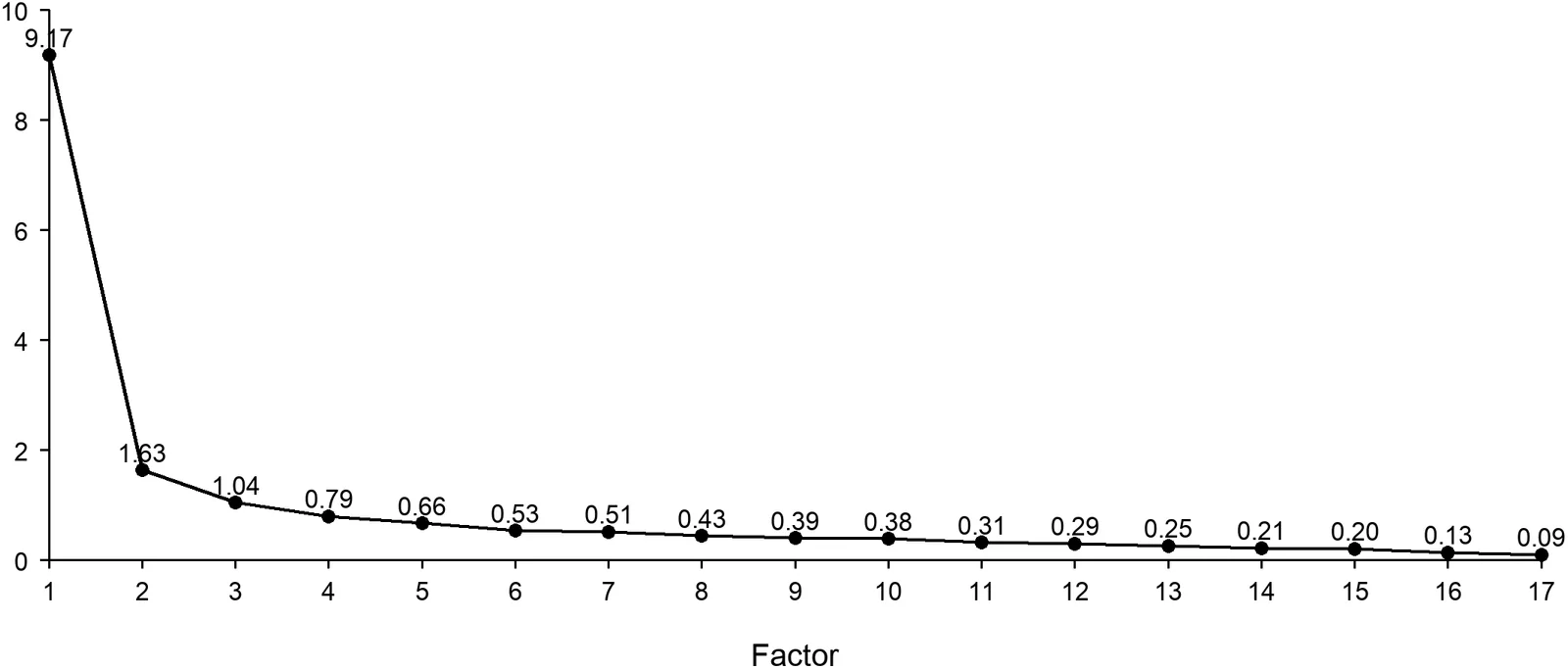
Scree plot.

It is essential to assess feasibility before conducting EFA. The KMO value was 0.944, and Bartlett’s test of sphericity yielded p < 0.001 (*χ²* = 5207.934, df = 136), indicating that the data were suitable for EFA. Principal component analysis was used to examine the component structure of the 17 secondary items. Three components with eigenvalues > 1 were retained. Based on the item content, these three components were interpreted as factors representing basic needs, HD treatment, and social support. Following orthogonal Varimax rotation, the variances explained by these three factors were 34.886%, 21.671%, and 13.126%, respectively, with a cumulative explained variance of 69.683%. In the scree plot, the eigenvalue of the fourth component fell below 1, and the curve subsequently flattened, indicating that later components contributed little additional explained variance. Thus, the scree plot supported the retention of three factors, consistent with the three dimensions proposed after two rounds of expert consultation. The scree plot is shown in Fig 1. Orthogonal Varimax rotation was used to clarify the correspondence between the factors and the items. The communalities of the secondary items were calculated, and all exceeded 0.40, indicating that the items were adequately represented by the retained factor solution. For further details, see Table 4.

**Table 4:** Exploratory factor analysis using principal component extraction and Varimax rotation (N = 395).

Items	Factor Loading Coefficient			Communality	Before Rotation			After Rotation		
	Factor 1	Factor 2	Factor 3		Eigenvalue	Explained Variation (%)	Cumulative (%)	Eigenvalue	Explained Variation (%)	Cumulative (%)
Item 1.1	**0.839**	0.378	0.216	0.893	9.173	53.957	53.957	5.931	34.886	34.886
Item 1.2	**0.775**	0.182	0.190	0.670						
Item 1.3	**0.780**	0.339	0.136	0.741						
Item 1.4	**0.720**	0.305	0.160	0.637						
Item 1.5	**0.808**	0.289	0.189	0.772						
Item 1.6	**0.740**	0.263	0.157	0.641						
Item 2.1	0.215	**0.801**	0.205	0.729	1.631	9.594	63.551	3.684	21.671	56.557
Item 2.2	0.315	**0.821**	0.208	0.817						
Item 2.3	0.493	**0.762**	0.122	0.838						
Item 2.4	0.553	**0.687**	0.120	0.792						
Item 2.5	0.344	**0.680**	0.097	0.591						
Item 2.6	0.404	**0.524**	0.050	0.440						
Item 2.7	0.327	**0.474**	−0.007	0.432						
Item 2.8	0.477	**0.754**	0.112	0.809						
Item 3.1	0.246	0.090	**0.768**	0.659	1.042	6.132	69.683	2.231	13.126	69.683
Item 3.2	0.357	0.170	**0.760**	0.734						
Item 3.3	−0.050	0.104	**0.859**	0.751						

**Convergent validity:** Convergent validity was supported by a strong negative correlation between HD-CDS and Barthel Index scores (*r* = −0.715, p < 0.001). The direction of this association was expected because higher HD-CDS scores indicate greater care dependency, whereas higher Barthel Index scores indicate greater functional independence. This correlation provides evidence of convergent validity but does not establish the Barthel Index as a gold-standard measure of hemodialysis-specific care dependency.

#### Reliability analysis.

The Cronbach’s α coefficient for the overall scale was 0.893 (95% CI: 0.87–0.908), and those for the three dimensions of basic needs, HD treatment, and social support were 0.921 (95% CI: 0.90–0.932), 0.878 (95% CI: 0.859–0.895), and 0.737 (95% CI: 0.689–0.779), respectively. The split-half coefficient for the overall scale was 0.885 (95% CI: 0.860–0.906), while the split-half coefficients for each dimension were 0.936 (95% CI: 0.922–0.947), 0.978 (95% CI: 0.973–0.982), and 0.755 (95% CI: 0.701–0.799). The overall scale and the basic-needs and HD-treatment dimensions showed satisfactory internal consistency. However, the social-support dimension did not meet the prespecified threshold of > 0.80, with a Cronbach’s α of 0.737 and a split-half coefficient of 0.755, indicating moderate preliminary internal consistency. Test–retest reliability was evaluated in the 39 participants included in the analysis using ICCs with a two-way mixed model with agreement. The ICC for the total score of the scale was 0.965 (95% CI: 0.933–0.982), with the ICCs for each dimension (95% CI) being 0.937 (0.880–0.967), 0.963 (0.959–0.989), and 0.970 (0.942–0.985), all within the prespecified range of 0.70–1.00, indicating excellent stability.

### Final scale

The HD-CDS we developed comprises 17 items across 3 dimensions (50 scoring criteria in total): basic needs, HD treatment, and social support (see S2 File).

### Correlates of HD-CDS scores

Significant differences in HD-CDS total scores were found across education levels, HD sessions per week, vascular access type, and vascular access site (all *p* < 0.001). Dialysis vintage and pre-dialysis blood pressure were significantly negatively correlated with care dependency (*p* < 0.001). No significant associations were observed for sex, age, or ultrafiltration rate (*p* > 0.05). Detailed results are presented in Table 5. More detailed statistical procedures are available in S4 Data.

**Table 5:** Associations between sociodemographic/clinical variables and HD-CDS total scores (N = 395).

Variables	Statistical method	Test value	*p value*
Sex	Independent samples *t* test	*t* = −0.281	0.779
Education	ANOVA	F = 24.585	< 0.001
Age	Pearson correlation	*r* = −0.013	0.795
Dialysis vintage (months)	Pearson correlation	*r* = −0.254	< 0.001
Ultrafiltration rate (%)	Pearson correlation	*r* = −0.086	0.087
Pre-HD SBP (mmHg)	Pearson correlation	*r* = −0.237	< 0.001
Pre-HD DBP (mmHg)	Pearson correlation	*r* = −0.309	< 0.001
Hemodialysis sessions per week	Kruskal–Wallis	H = 89.753	< 0.001
Vascular access type	Kruskal–Wallis	H = 78.088	< 0.001
Vascular access site	Kruskal–Wallis	H = 78.063	< 0.001

## Discussion

This study developed the HD-CDS and provided preliminary evidence of its content validity, internal consistency, test–retest reliability, and exploratory structural validity in a single-center sample. The three dimensions capture the unique dependency profile of patients undergoing HD: basic needs reflect compromised daily functions; HD treatment addresses procedure‑specific care; and social support covers emotional and informational needs. This may be related to the fact that the scale was developed based on Henderson’s theory of basic human needs, along with literature reviews, NGT, and Delphi expert consultations. Although the overall scale showed satisfactory internal consistency, the lower coefficients for the social-support dimension warrant further examination in an independent sample. The HD-CDS showed a strong negative correlation with the Barthel Index (*r* = −0.715, p < 0.001). The Barthel Index assesses activities of daily living and is conceptually related to care dependency, but it is not a gold-standard measure of hemodialysis-specific care dependency. Therefore, the observed correlation should be interpreted as evidence of convergent validity rather than definitive criterion validity. Future studies should validate the HD‑CDS against other measures, such as nursing workload or the direct observation of dependency.

Regarding the relationships between sociodemographic/clinical variables and HD-CDS scores, several significant findings emerged. Patients with lower education levels, more frequent HD sessions, and temporary or more complex vascular access types tended to have higher care dependency scores (all *p* < 0.001). These associations likely reflect that greater disease burden and treatment intensity increase the need for nursing support. Notably, dialysis vintage and pre-dialysis blood pressure were negatively correlated with care dependency (*p* < 0.001). Patients with more experience with dialysis may develop better self-management skills, leading to reduced dependency over time. Lower pre-dialysis blood pressure, which often indicates poorer cardiovascular stability or higher ultrafiltration needs, might paradoxically be associated with greater dependency. No significant associations were found for sex, age, or ultrafiltration rate (*p* > 0.05), suggesting that care dependency in this population is more strongly driven by treatment-related and cumulative disease factors rather than by basic demographic characteristics. These exploratory associations require confirmation in independent samples before they can be used to guide targeted nursing interventions.

The HD-CDS may provide a structured framework for describing care dependency across basic needs, HD treatment, and social support. In China, limited nursing resources and the growing number of patients undergoing HD highlight the need for systematic assessment of care dependency [38]. Existing regulations specify staffing ratios for conventional HD and continuous renal replacement therapy [39,40]. China’s 14th Five-Year Plan for nursing development also emphasizes refined nursing-process management [41]. However, the present study did not evaluate nursing workload, staffing allocation, clinical effectiveness, falls, infections, or other patient-safety outcomes. Therefore, its potential applications in individualized care planning and resource allocation should be regarded as hypotheses for future evaluation rather than established clinical benefits.

The HD-CDS was designed with potential clinical applicability in mind. There are two primary methods for classifying patients according to their nursing dependency: the prototype system and the factor system [42]. The prototype system is based on a broad description of the typical characteristics of patient categories, which is usually quantified in terms of daily nursing hours. For patients undergoing HD, the nursing hours are typically around 4 h. However, patients with the same treatment duration have identical nursing hours, making it impossible to differentiate their levels of nursing dependency. Thus, this study adopted the factor system approach to set items, classifying patients according to the specific nursing measures they require. We also referred to the scoring methods of the NPDS [43] and CUDYR-DIAL [44] scales, assigning scores according to the difficulty, frequency, and intensity of the nursing measures, with the aim of supporting future clinical evaluation. This approach was intended to reduce potential score discrepancies across assessors; however, inter-rater reliability was not evaluated in the present study. Additionally, during two rounds of expert consultations, the ordering and scoring settings of the items were adjusted based on importance ratings and expert opinions, further improving the scale’s practicality.

### Limitations

This study has several limitations. First, the geographical distribution of experts participating in the Delphi consultation was relatively limited, and the Kendall’s W values for the first- and second-level items indicated only fair to moderate expert agreement. Second, all participants were recruited from a single tertiary hospital in Changzhou, China. The patient case mix, staffing patterns, nurse training, care workflows, and local clinical practices may differ from those in other hospitals, regions, and health-care systems. Therefore, the present findings should be interpreted as preliminary evidence and cannot yet be generalized beyond the study setting. Multicenter studies involving different regions and facility levels, together with independent structural validation, are required to evaluate the external validity and transportability of the HD-CDS. Third, the detailed scoring criteria may be time-consuming for first-time users. Finally, the present study did not evaluate inter-rater reliability, nursing workload, staffing decisions, clinical effectiveness, or patient-safety outcomes. These aspects should be examined before routine clinical implementation.

## Conclusion

The HD-CDS demonstrated promising preliminary psychometric properties in a single-center sample and may provide a useful framework for assessing care dependency among patients undergoing hemodialysis. Further multicenter studies, including independent structural validation and inter-rater reliability assessment, are required before routine clinical implementation.

## Supporting information

S1 Data Participant-level demographic, clinical, HD-CDS, and Barthel Index data for 395 participants used in the psychometric analyses. (XLSX)

S2 Data Four-point relevance ratings from six experts used to calculate the I-CVI and S-CVI. (XLSX)

S3 Data First and second HD-CDS total and dimension scores for 39 participants used to assess test–retest reliability. (XLSX)

S4 Data Detailed statistical analyses of associations between participant characteristics and HD-CDS total scores. (XLSX)

S1 File The Delphi Process for the HD-CDS. This file details the entire Delphi process, including the initial item pool, the revised items after each of the two Delphi rounds, and the rationale behind all the modifications.
(PDF)

S2 File Final HD-CDS after Psychometric Evaluation. This file presents the final version of the HD-CDS, including all items and scoring instructions, as developed and preliminarily evaluated in the present psychometric study.
(DOCX)

## Abbreviations

HD-CDS: Hemodialysis patient care dependency scale
NGT: Nominal group technique
Cr: Authority coefficient
Cs: Familiarity coefficient
Ca: Judgment basis coefficient
Kendall’s W: Kendall’s coefficients of concordance
CV: Coefficient of variation
I-CVI: Item-level content validity index
S-CVI: Scale-level content validity index
EFA: Exploratory factor analysis
KMO: Kaiser–Meyer–Olkin
SPSS: Statistical Package for Social Sciences
ICC: Intraclass correlation coefficient
INICIARE: Inventario del NIvel de Cuidados mediante Indicadores de Resultados de Enfermería (Spanish)
CUDYR-DIAL: Categorización de usuario según dependencia y riesgo en unidades de hemodiálisis (Spanish)
NPDS: Northwick Park Dependency Score
