# Supplementary material for: Development and preliminary psychometric evaluation of the hemodialysis patient care dependency scale (HD-CDS)
Source: PLoS One. 2026 Sep 15;21(9):e0358118. doi: 10.1371/journal.pone.0358118 (PMC13577502; doi:10.1371/journal.pone.0358118)
Supplement: Supplementary file 1 [file pone.0358118.s005.pdf]

Additional file 1.

The Delphi process of the HD-CDS

Initial Items	First round of revisions	Items After Round 1 Delphi	Second round of revisions	Items After Round 2 Delphi
1. Basic needs	Mark the score according to expert advice	1. Basic needs (0-19 points)	No adjustments were made	1. Basic needs (0-19 points)
1.1 Maintain normal respiration	Mark the score according to expert advice	1.1 Maintain normal respiration (0-3 points)	No adjustments were made	1.1 Maintain normal respiration (0-3 points)
1.1.1 Need to rely on advanced respiratory support system to maintain respiration (e.g., ECMO, ventilator, etc.) (3 points)	No adjustments were made	1.1.1 Need to rely on advanced respiratory support system to maintain respiration (e.g., ECMO, ventilator, etc.) (3 points)	Linguistic optimization	1.1.1 Relies on advanced respiratory support system to maintain respiration (e.g., ECMO, ventilator) (3 points)
1.1.2 High flow oxygen is required (2 points)	No adjustments were made	1.1.2 High flow oxygen is required (2 points)	No adjustments were made	1.1.2 High flow oxygen is required (2 points)
1.1.3 Low flow oxygen is required (1 point)	No adjustments were made	1.1.3 Low flow oxygen is required (1 point)	No adjustments were made	1.1.3 Low flow oxygen is required (1 point)
1.1.4 No oxygen is required (0 points)	Importance score <3.5, deleted	-	No adjustments were made	-
1.2 Dietary care	No adjustments were made	1.2 Dietary care (0-3 points)	No adjustments were made	1.2 Dietary care (0-3 points)
1.2.1 Requires parenteral nutrition support (3 points)	No adjustments were made	1.2.1 Requires parenteral nutrition support (3 points)	No adjustments were made	1.2.1 Requires parenteral nutrition support (3 points)
1.2.2 Requires nasogastric feeding (2 points)	No adjustments were made	1.2.2 Requires nasogastric feeding (2 points)	No adjustments were made	1.2.2 Requires nasogastric feeding (2 points)
1.2.3 Can eat by mouth, but need help (1 point)	No adjustments were made	1.2.3 Can eat orally, but with assistance (1 point)	Linguistic optimization	1.2.3 Can eat orally with assistance (1 point)
1.2.4 Can eat by mouth by themselves (0 points)	Importance score <3.5, deleted	-	-	-

Additional file 1.

The Delphi process of the HD-CDS

Initial Items	First round of revisions	Items After Round 1 Delphi	Second round of revisions	Items After Round 2 Delphi
1.3 Excretion care	Adjust the ranking to 1.5 according to the importance score, and mark the score according to the expert's suggestion	1.3 Help to move or transfer (0-4 points)	Linguistic optimization	1.3 Assistance with movement or transfer (0-4 points)
1.3.1 The patient needs to be discharged through the catheter and fistula (4 points)	According to the importance score, the order was adjusted to 1.5.1, and "and" was changed to "or" according to expert suggestions	1.3.1 Relies on trolley for transfer (4 points)	No adjustments were made	1.3.1 Relies on trolley for transfer (4 points)
1.3.2 The patient has normal excretion but needs to use a pad or diaper due to urinary incontinence or fecal incontinence (3 points)	Adjusted the order to 1.5.2 according to the importance score	1.3.2 Need to rely on wheelchair transfer (3 points)	Linguistic optimization	1.3.2 Relies on wheelchair for transfer (3 points)
1.3.3 The patient's normal excretion is in a suitable container (toilet or chamber pot) and needs to be assisted by a person (2 points)	Adjusted the order to 1.5.3 according to the importance score	1.3.3 Need to rely on a cane or support to walk (2 points)	The score is optimized to 3 points according to the importance score and Linguistic optimization	1.3.3 Relies on a cane or other support to walk (3 points)
1.3.4 The patient needs to go to the toilet with help and supervision (1 point)	According to the importance score, it was adjusted and ranked to 1.5.4. Meanwhile, according to the expert's suggestion, it was modified to "get out of bed and go to the toilet"	1.3.4 The patient can walk or move without help, but needs to be reminded or supervised safety (1 point)	No adjustments were made	1.3.4 The patient can walk or move without help, but needs to be reminded or supervised for safety (1 point)

Additional file 1.

The Delphi process of the HD-CDS

Initial Items	First round of revisions	Items After Round 1 Delphi	Second round of revisions	Items After Round 2 Delphi
1.3.5 The patient can go to the toilet without help (0 points)	Importance score <3.5, deleted	-	-	-
1.4 Keep a comfortable position	Mark the score according to expert advice	1.4 Maintain a comfortable position (0-3 points)	No adjustments were made	1.4 Maintain a comfortable position (0-3 points)
1.4.1 The patient needs physical restraint due to the need of hemodialysis treatment (3 points)	No adjustments were made	1.4.1 The patient needs physical restraint due to the need of hemodialysis treatment (3 points)	Linguistic optimization	1.4.1 The patient needs physical restraint due to the need for haemodialysis treatment (3 points)
1.4.2 The patient is forced to adopt a position due to the need for hemodialysis treatment or to improve pain (2 points)	No adjustments were made	1.4.2 The patient is forced to adopt a position due to the need for hemodialysis treatment or to improve pain (2 points)	Linguistic optimization	1.4.2 The patient is forced to adopt a position due to the need for haemodialysis treatment or to alleviate pain (2 points)
1.4.3 The patient has no consciousness to change the position at regular time, and needs to be assisted to change the position (1 point)	No adjustments were made	1.4.3 The patient has no consciousness to change the position at regular time, and needs to be assisted to change the position (1 point)	Linguistic optimization	1.4.3 The patient is not aware that their position needs to change at a regular time and needs assistance with changing positions (1 point)
1.4.4 Patients can change their position by themselves (0 points)	Importance score <3.5, deleted	-	-	-
1.5 Help to move or transfer	Adjust the ranking to 1.3 according to the importance score, and mark the score according to expert suggestions	1.5 Excretion care (0-4 points)	No adjustments were made	1.5 Excretion care (0-4 points)

Additional file 1.

The Delphi process of the HD-CDS

Initial Items	First round of revisions	Items After Round 1 Delphi	Second round of revisions	Items After Round 2 Delphi
1.5.1 The patient was unable to walk and was transported to the hemodialysis room on a trolley. Full assistance was needed to help him get into bed (3 points)	According to the importance score, the ranking was adjusted to 1.3.1. According to the expert's suggestion, the expression was simplified and the score was adjusted	1.5.1 The patient needs to be discharged through a catheter or fistula (4 points)	No adjustments were made	1.5.1 The patient needs to be discharged through a catheter or fistula (4 points)
1.5.2 The patient needs assistance to transfer from the wheelchair to the bed (2 points)	According to the importance score, it was adjusted and ranked to 1.3.2. According to the expert's suggestion, the expression was simplified and one item 1.3.3 "relying on crutches or handrails" was added, and the score was adjusted	1.5.2 The patient has normal excretion but needs to use a pad or diaper due to urinary incontinence or fecal incontinence (3 points)	Revise the expression according to expert advice and Linguistic optimization	1.5.2 The patient has normal excretory function but needs to use a pad or diaper due to urinary or faecal incontinence (3 points)
1.5.3 The patient can walk or move without help, but needs to be reminded and supervised safely (1 point)	According to the importance score, the order was adjusted to 1.3.4, and "and" was changed to "or" according to expert suggestions	1.5.3 The patient's normal excretion is in a suitable container (toilet or chamber pot) and needs assistance (2 points)	Linguistic optimization	1.5.3 The patient can excrete normally in a suitable container (toilet or chamber pot) with assistance (2 points)
1.5.4 The patient can walk or move without any assistance (0 points)	Importance score <3.5, deleted	1.5.4 The patient can get out of bed to go to the toilet, but needs help and supervision (1 point)	No adjustments were made	1.5.4 The patient can get out of bed to go to the toilet, but needs help and supervision (1 point)
1.6 Choose the right clothes	Mark the score according to expert advice	1.6 Choose the right clothes (0-2 points)	Linguistic optimization	1.6 Choosing the right clothes (0-2 points)

Additional file 1.

The Delphi process of the HD-CDS

Initial Items	First round of revisions	Items After Round 1 Delphi	Second round of revisions	Items After Round 2 Delphi
1.6.1 Exposure of the limb or hemodialysis catheter on the side of the arteriovenous fistula due to clothing selected by the patient (2 points)	No adjustments were made	1.6.1 Exposure of the limb or hemodialysis catheter on the side of the arteriovenous fistula due to clothing selected by the patient (2 points)	Linguistic optimization	1.6.1 Clothing selected by the patient does not allow exposure of the limb or haemodialysis catheter on the side of the arteriovenous fistula (2 points)
1.6.2 The clothing selected by the patient does not affect the exposure of the limb on the side of the arteriovenous fistula or the hemodialysis catheter, but the dressing action cannot be completed by himself (1 point)	No adjustments were made	1.6.2 The clothing selected by the patient does not affect the exposure of the limb on the side of the arteriovenous fistula or the hemodialysis catheter, but the dressing action cannot be completed by himself (1 point)	Linguistic optimization	1.6.2 Clothing selected by the patient does not affect exposure of the limb on the side of the arteriovenous fistula or haemodialysis catheter, but the action of dressing cannot be completed by themself (1 point)
1.6.3 The clothing selected by the patient does not affect the exposure of the limb on the side of the arteriovenous fistula or the hemodialysis catheter, and the patient can complete the action of undressing and dressing by himself (0 points)	Importance score <3.5, deleted	-	-	-
2. Hemodialysis	Mark the score according to expert advice	2. Hemodialysis (5-24 points)	Linguistic optimization	2. Haemodialysis (5-24 points)
2.1 Monitoring of vital signs	Mark the score according to expert advice	2.1 Monitoring of vital signs (1-4 points)	No adjustments were made	2.1 Monitoring of vital signs (1-4 points)
2.1.1 The interval time of vital sign monitoring in hemodialysis treatment is less than 15min (4 points)	No adjustments were made	2.1.1 The interval time of vital sign monitoring in hemodialysis treatment is less than 15min (4 points)	Linguistic optimization	2.1.1 Time interval of vital sign monitoring during haemodialysis is less than 15 min (4 points)

Additional file 1.

The Delphi process of the HD-CDS

Initial Items	First round of revisions	Items After Round 1 Delphi	Second round of revisions	Items After Round 2 Delphi
2.1.2 15min ≤ interval time of vital sign monitoring during hemodialysis treatment <30min (3 points)	No adjustments were made	2.1.2 15min ≤ interval time of vital sign monitoring during hemodialysis treatment <30min (3 points)	Linguistic optimization	2.1.2 Time interval of vital sign monitoring during haemodialysis is ≤15 min but <30 min (3 points)
2.1.3 30min ≤ interval time of vital sign monitoring during hemodialysis treatment <60min (2 points)	No adjustments were made	2.1.3 30min ≤ interval time of vital sign monitoring during hemodialysis treatment <60min (2 points)	Linguistic optimization	2.1.3 Time interval of vital sign monitoring during haemodialysis is ≤30 min but <60 min (2 points)
2.1.4 The interval time of vital sign monitoring in hemodialysis treatment =60min (1 minute)	No adjustments were made	2.1.4 The interval time of vital sign monitoring in hemodialysis treatment =60min (1 minute)	Linguistic optimization	2.1.4 Time interval of vital sign monitoring during haemodialysis =60 min (1 point)
2.2 Monitoring of intake or weight gain	According to the importance score, adjust the ranking to 2.4, and mark the score according to expert suggestions	2.2 Invasive nursing operations (e.g., connection and disconnection of hemodialysis catheter and hemodialysis machine, placement of endostatic puncture needle, etc.) (1-3 points)	Linguistic optimization	2.2 Invasive nursing operations (e.g., connection and disconnection of haemodialysis catheter and machine, placement of end puncture needle) (1-3 points)
2.2.1 The total ultrafiltration amount or ultrafiltration rate of hemodialysis treatment should be determined by monitoring the liquid inflow and outflow (2 points)	Rank 2.4.1 according to the importance score	2.2.1 The nurse needs to perform three or more invasive operations during dialysis (3 points)	According to expert advice, the expression was simplified and "during dialysis" was changed to "during hemodialysis treatment"	2.2.1 The nurse performs three or more invasive operations during dialysis (3 points)

Additional file 1.

The Delphi process of the HD-CDS

Initial Items	First round of revisions	Items After Round 1 Delphi	Second round of revisions	Items After Round 2 Delphi
2.2.2 Weighing is required to determine the total ultrafiltration volume of hemodialysis treatment (1 point)	Rank 2.4.2 according to the importance score	2.2.2 The nurse performed two invasive operations during dialysis (2 points)	According to expert advice, "during dialysis" was changed to "during hemodialysis treatment"	2.2.2 The nurse performs two invasive operations during dialysis (2 points)
		2.2.3 The nurse performed an invasive operation during dialysis (1 point)	According to expert advice, "during dialysis" was changed to "during hemodialysis treatment"	2.2.3 The nurse performs one invasive operation during dialysis (1 point)
2.3 Risk monitoring	According to the importance score, adjust the ranking to 2.8, and mark the score according to expert suggestions	2.3 Nursing of the puncture point of internal fistula or blood dialysis catheter (1-3 points)	No adjustments were made	2.3 Nursing of the puncture point of internal fistula or blood dialysis catheter (1-3 points)
2.3.1 The patient has had previous accidents (catheter slippage, needle slippage, fall, fall from bed, loss of consciousness, etc.) (3 points)	According to the importance score, the order is adjusted to 2.8.1 and the score is adjusted	2.3.1 During hemodialysis, to avoid bleeding at the puncture point of the internal fistula or continuous exudation around the catheter, disinfection or dressing coverage should be performed on the skin at the puncture point of the internal fistula or around the catheter, and sterile operation should be performed at least once (3 points)	Linguistic optimization	2.3.1 During haemodialysis, to avoid the continuous exudation of blood or exudate around the puncture point of the fistula or catheter, the skin around the puncture point of the fistula or catheter needs to be disinfected or covered with a sterile operation at least once (3 points)

Additional file 1.

The Delphi process of the HD-CDS

Initial Items	First round of revisions	Items After Round 1 Delphi	Second round of revisions	Items After Round 2 Delphi
2.3.2 The patient has no previous accident, but has high risk factors (physical or cognitive limitations, over 70 years old, etc.) (2 points)	According to the importance score, the order is adjusted to 2.8.2 and the score is adjusted	2.3.2 During hemodialysis, to promote the healing of the internal fistula puncture after needle removal, gauze or cotton ball was used to apply pressure and bandage the puncture point of the internal fistula for 3 times or more, and the technique used was clean operation (2 points)	Linguistic optimization	2.3.2 During haemodialysis, to promote healing of the internal fistula puncture after needle removal, gauze or a cotton ball is used to apply pressure and the puncture point of the internal fistula is bandaged 3 times or more using a clean operation technique (2 points)
2.3.3 The patient was conscious and cooperative with the treatment (1 point)	Importance score <3.5, deleted	2.3.3 During hemodialysis, to promote the healing of the internal fistula puncture after needle removal or to maintain the cleanliness of the catheter opening, gauze or cotton ball was used for covering or bandaging 1-2 times, and the technique used was clean operation (1 point)	Linguistic optimization	2.3.3 During haemodialysis, to promote the healing of the internal fistula puncture after needle removal or to maintain the cleanliness of the catheter opening, gauze or cotton ball is used to cover or bandage the area 1-2 times using a clean operation technique (1 point)
2.4 Adjustment of original hemodialysis prescription (prescription content includes: conductivity, temperature, total ultrafiltration, dialysis mode, anticoagulant dose, replacement fluid dose, etc.)	According to the importance score, adjust the ranking to 2.6, and mark the score according to expert suggestions	2.4 Monitoring of intake or weight gain (1-2 points)	Linguistic optimization	2.4 Monitoring intake or weight gain (1-2 points)

Additional file 1.

The Delphi process of the HD-CDS

Initial Items	First round of revisions	Items After Round 1 Delphi	Second round of revisions	Items After Round 2 Delphi
2.4.1 Original prescription changed 8 times or more due to various reasons such as blood pressure change, spasm, allergy, elevated pressure of extracorporeal circulation pipeline, bleeding, etc. (4 points)	According to the importance score, the order is adjusted to 2.6.1 and the score is adjusted	2.4.1 The total ultrafiltration amount or ultrafiltration rate of hemodialysis treatment should be determined by monitoring the liquid inflow and outflow (2 points)	Linguistic optimization	2.4.1 The total ultrafiltration volume or rate of haemodialysis should be determined by monitoring the liquid inflow and outflow (2 points)
2.4.2 The original prescription was changed 5 to 7 times due to various reasons such as blood pressure change, spasm, allergy, elevated pressure of extracorporeal circulation pipeline, bleeding, etc. (3 points)	According to the importance score, the order is adjusted to 2.6.2 and the score is adjusted	2.4.2 Weighing is required to determine the total ultrafiltration volume of hemodialysis treatment (1 point)	Linguistic optimization	2.4.2 Weighing is required to determine the total ultrafiltration volume of haemodialysis (1 point)
2.4.3 Original prescription changed 1 to 4 times due to various reasons such as blood pressure change, spasm, allergy, elevated pressure of extracorporeal circulation pipeline, bleeding, etc. (2 points)	According to the importance score, the order is adjusted to 2.6.3 and the score is adjusted	-	-	-
2.4.4 No changes in the original prescription due to various reasons (1 point)	Importance score <3.5, deleted	-	-	-
2.5 Management of drug therapy	Mark the score according to expert advice	2.5 Management of drug therapy (0-3 points)	Linguistic optimization	2.5 Drug therapy management (0-3 points)

Additional file 1.

The Delphi process of the HD-CDS

Initial Items	First round of revisions	Items After Round 1 Delphi	Second round of revisions	Items After Round 2 Delphi
2.5.1. The hemodialysis treatment requires the use of three or more drugs and one or more drugs need to be controlled by an infusion pump or micro pump (3 points)	According to expert advice, it was changed to "in hemodialysis treatment, there are more than one drug that needs strict control of the speed"	2.5.1 In hemodialysis treatment, there are more than one drugs that need strict control of the speed (3 points)	Linguistic optimization	2.5.1 The administration speed of more than one drug needs to be strictly controlled during haemodialysis treatment (3 points)
2.5.2 Use of three or fewer drugs in hemodialysis treatment and at least one drug needs to be controlled by infusion pump or micro pump (2 points)	According to expert advice, it should be changed to "three or more drugs are needed in hemodialysis treatment but the speed does not need to be strictly controlled"	2.5.2 Use of three or more drugs in hemodialysis treatment without strict control of the speed (2 points)	Linguistic optimization	2.5.2 The administration of three or more drugs during haemodialysis that do not require strict control of the speed (2 points)
2.5.3 Use of three or fewer drugs in hemodialysis treatment without the need for infusion pump or micro pump control (1 point)	According to expert advice, it should be changed to "use 1 or 2 drugs in hemodialysis treatment and do not need strict control of the speed"	2.5.3 Use of 1 to 2 drugs in hemodialysis treatment without strict control of the speed (1 point)	Linguistic optimization	2.5.3 Use of 1 to 2 drugs during haemodialysis that do not require strict control of the speed (1 point)
2.6 Invasive nursing operations (e.g., connection and disconnection of hemodialysis catheter and hemodialysis machine, insertion of endostatic puncture needle, etc.)	According to the importance score, adjust the ranking to 2.2, and mark the score according to expert suggestions	2.6 Adjustment of original hemodialysis prescription (prescription content includes: conductivity, temperature, total ultrafiltration, dialysis mode, anticoagulant dose, replacement fluid dose, etc.) (0-3 points)	Linguistic optimization	2.6 Adjustment of the original haemodialysis prescription (including conductivity, temperature, total ultrafiltration volume, dialysis mode, anticoagulant dose, replacement fluid dose, etc.) (0-3 points)

Additional file 1.

The Delphi process of the HD-CDS

Initial Items	First round of revisions	Items After Round 1 Delphi	Second round of revisions	Items After Round 2 Delphi
2.6.1 The nurse needs to perform three or more invasive operations during dialysis (3 points)	Rank 2.2.1 according to the importance score	2.6.1 Original prescription changed 8 times or more due to various reasons such as blood pressure change, spasm, allergy, elevated pressure of extracorporeal circulation pipeline, bleeding, etc. (3 points)	According to the expert advice, "change 8 times or more" is changed to "change frequency ≥2 times/hour"	2.6.1Original prescription changed 8 times or more due to various reasons including change in blood pressure, spasm, allergy, elevated pressure of extracorporeal circulation pipeline, bleeding, etc. (3 points)
2.6.2 The nurse performed two invasive operations during dialysis (2 points)	Rank 2.2.2 according to the importance score	2.6.2 The original prescription was changed 5 to 7 times due to various reasons such as blood pressure change, spasm, allergy, elevated pressure of extracorporeal circulation pipeline, bleeding, etc. (2 points)	According to expert advice, "change 8 times or more" was changed to "change frequency of 1 to 2 times/hour"	2.6.2 Original prescription was changed 5 to 7 times due to various reasons including change in blood pressure, spasm, allergy, elevated pressure of extracorporeal circulation pipeline, bleeding, etc. (2 points)
2.6.3 The nurse performed an invasive operation during dialysis (1 point)	Rank 2.2.3 according to the importance score	2.6.3 Original prescription changed 1 to 4 times due to various reasons such as blood pressure change, spasm, allergy, elevated pressure of extracorporeal circulation pipeline, bleeding, etc. (1 point)	According to the expert advice, "change 8 times or more" is changed to "change frequency ≤1 time/hour"	2.6.3 Original prescription changed 1 to 4 times due to various reasons including change in blood pressure, spasm, allergy, elevated pressure of extracorporeal circulation pipeline, bleeding, etc. (1 point)
2.7 Complications of vascular access	Mark the score according to expert advice	2.7 Complications of vascular access (1-4 points)	According to expert advice, it was revised to "vascular pathway care"	2.7 Vascular pathway care (1-4 points)
2.7.1 The fistula is difficult to puncture and requires an experienced nurse to puncture or the catheter needs thrombolysis (3 points)	Adjust the score according to expert advice	2.7.1 The fistula is difficult to puncture and requires an experienced nurse to puncture or the catheter needs thrombolysis (4 points)	Linguistic optimization	2.7.1 The fistula is difficult to puncture and requires an experienced nurse or thrombolysis in the catheter needs to be performed (4 points)

Additional file 1.

The Delphi process of the HD-CDS

Initial Items	First round of revisions	Items After Round 1 Delphi	Second round of revisions	Items After Round 2 Delphi
2.7.2 There are complications of internal fistula (hematoma, collapse, insufficient blood flow, infection, etc.) or complications of catheter (insufficient blood flow, catheter rupture, thrombosis, infection, etc.) (2 points)	Adjust the score according to expert advice	2.7.2 There are complications of internal fistula (hematoma, collapse, insufficient blood flow, infection, etc.) or complications of catheter (insufficient blood flow, catheter rupture, thrombosis, infection, etc.) (3 points)	Linguistic optimization	2.7.2 Complications with the internal fistula (haematoma, collapse, insufficient blood flow, infection, etc.) or catheter (insufficient blood flow, catheter rupture, thrombosis, infection, etc.) (3 points)
2.7.3 The method of internal fistula puncture was eye-piercing or catheter without complications (2 points)	No adjustments were made	2.7.3 The method of internal fistula puncture was eye-piercing or catheter without complications (2 points)	Linguistic optimization	2.7.3 Internal fistula puncture performed in the buttonhole or catheter placement without complications (2 points)
2.7.4 No complications of internal fistula and rope ladder puncture (1 point)	No adjustments were made	2.7.4 No complications of internal fistula and rope ladder puncture (1 point)	Linguistic optimization	2.7.4 No complications of internal fistula rope ladder puncture (1 point)
2.8 Nursing of puncture point of internal fistula or blood dialysis catheter	According to the importance score, adjust the ranking to 2.3, and mark the score according to expert suggestions	2.8 Risk monitoring (0-2 points)	No adjustments were made	2.8 Risk monitoring (0-2 points)
2.8.1 During hemodialysis, to avoid continuous exudation of blood or exudate around the puncture point of the fistula or around the catheter, disinfect or cover the skin around the puncture point of the fistula or around the catheter with sterile operation 1-3 times (3 points)	According to the importance score, the ranking was adjusted to 2.3.1, and "1-3 times" was changed to "≥1 time" according to expert suggestions	2.8.1 The patient has had previous accidents (catheter slippage, needle slippage, fall, fall from bed, loss of consciousness, etc.) (2 points)	No adjustments were made	2.8.1 The patient has had previous accidents (catheter slippage, needle slippage, fall, fall from bed, loss of consciousness, etc.) (2 points)

Additional file 1.

The Delphi process of the HD-CDS

Initial Items	First round of revisions	Items After Round 1 Delphi	Second round of revisions	Items After Round 2 Delphi
2.8.2 During hemodialysis, to promote the healing of the internal fistula puncture after needle removal, gauze or cotton ball was used to apply pressure and bandage the puncture point of the internal fistula for 3 times or more, and the technique used was clean operation (2 points)	Rank 2.3.2 according to the importance score	2.8.2 The patient has no previous accident, but has high risk factors (physical or cognitive limitations, over 70 years old, etc.) (1 point)	Linguistic optimization	2.8.2 The patient has had no previous accidents but has high risk factors (physical or cognitive limitations, more than 70 years of age, etc.) (1 point)
2.8.3 During hemodialysis, to promote the healing of the internal fistula puncture after needle removal or to maintain the cleanliness of the catheter opening, cover or bandage with gauze or cotton ball for 1-2 times, and the technique used is clean operation (1 point)	Rank 2.3.3 according to the importance score	-	-	-
3. Health care	Merge and annotate the scores according to expert advice	3. Social support (0-7 points)	No adjustments were made	3. Social support (0-7 points)
3.1 Promoting health	Revise the expression according to expert advice	3.1 Decision support (0-3 points)	Linguistic optimization	3.1 Support decisions (0-3 points)
3.1.1 Failure to actively seek help when complications or adverse reactions occur (4 points)	Adjust the score	3.1.1 Failure to actively seek help when complications or adverse reactions occur (3 points)	Linguistic optimization	3.1.1 The patient fails to actively seek help when complications or adverse reactions occur (3 points)

Additional file 1.

The Delphi process of the HD-CDS

Initial Items	First round of revisions	Items After Round 1 Delphi	Second round of revisions	Items After Round 2 Delphi
3.1.2 Actively seek help from medical staff when complications or adverse reactions occur, but cannot completely follow the guidance of medical staff (3 points)	Adjust the score	3.1.2 Actively seek help from medical staff when complications or adverse reactions occur, but cannot completely follow the guidance of medical staff (2 points)	Linguistic optimization	3.1.2 The patient actively seeks help from medical staff when complications or adverse reactions occur but cannot completely follow their guidance (2 points)
3.1.3 Actively seek help from medical staff when complications or adverse reactions occur, and be able to follow the guidance of medical staff (2 points)	Adjust the score	3.1.3 Actively seek help from medical staff when complications or adverse reactions occur, and can follow the guidance of medical staff (1 point)	Linguistic optimization	3.1.3 The patient actively seeks help from medical staff when complications or adverse reactions occur and can follow their guidance (1 point)
3.1.4 Actively seek the possibility of kidney transplantation (1 point)	Importance score <3.5, deleted	-	-	-
4. Social support	Merge according to experts	-	-	-
4.1 Emotional support	Merge and annotate the scores according to expert advice	3.2 Emotional support (0-3 points)	No adjustments were made	3.2 Emotional support (0-3 points)
4.1.1 Have suicidal behavior or suicidal tendency (4 points)	Adjust the score	3.2.1 Have suicidal behavior or suicidal tendency (3 points)	Linguistic optimization	3.2.1 The patient has suicidal behaviours or tendencies (3 points)
4.1.2 Insult or quarrel with family members or medical staff (3 points)	According to the importance score, the order is adjusted to 3.2.3 and the score is adjusted	3.2.2 There are anxiety, tension, depression, sadness, pain, depression and other emotions (2 points)	Linguistic optimization	3.2.2 The patient has anxiety, tension, depression, sadness, pain, depression and other emotions (2 points)
4.1.3 There are anxiety, tension, depression, sadness, pain, depression and other emotions (2 points)	According to the importance score, the order is adjusted to 3.2.2 and the score is adjusted	3.2.3 Insulting or quarreling with family members or medical staff (1 point)	Linguistic optimization	3.2.3 The patient insults or quarrels with family members or medical staff (1 point)

Additional file 1.

The Delphi process of the HD-CDS

Initial Items	First round of revisions	Items After Round 1 Delphi	Second round of revisions	Items After Round 2 Delphi
4.1.4 Emotional stability and acceptance of current condition (1 point)	Importance score <3.5, deleted	-	-	-
4.2 Material support	Merge and annotate the scores according to expert advice	3.3 Material support (0-1 point)	No adjustments were made	3.3 Material support (0-1 point)
4.2.1 The patient's family is unwilling to provide more material and economic help for the treatment of the disease (2 points)	Adjust the score	3.3.1 The patient's family is unwilling to provide more material and economic help for the treatment of the disease (1 point)	Revise the expression according to expert advice	3.3.1 The patient's family is unwilling to provide more material and economic assistance for disease treatment (1 point)
4.2.2 The family of the patient is more active in the treatment of the disease (1 point)	Importance score <3.5, deleted	-	-	-
