# Supplementary material for: Development and preliminary psychometric evaluation of the hemodialysis patient care dependency scale (HD-CDS)
Source: PLoS One. 2026 Sep 15;21(9):e0358118. doi: 10.1371/journal.pone.0358118 (PMC13577502; doi:10.1371/journal.pone.0358118)
Supplement: Supplementary file 2 [file pone.0358118.s006.docx]

| dimension | Items | scoring criteria | score | Dimensional total score |
| --- | --- | --- | --- | --- |
| 1. Basic needs (0-19 points) | 1.1 Maintain normal respiration (0-3 points) | 1.1.1 Need to rely on advanced respiratory support system to maintain respiration (e.g., ECMO, ventilator, etc.) (3 points) |  |  |
|  |  | 1.1.2 High flow oxygen is required (2 points) |  |  |
|  |  | 1.1.3 Low flow oxygen is required (1 point) |  |  |
|  | 1.2 Dietary care (0-3 points) | 1.2.1 Requires parenteral nutrition support (3 points) |  |  |
|  |  | 1.2.2 Requires nasogastric feeding (2 points) |  |  |
|  |  | 1.2.3 Can eat orally, but with assistance (1 point) |  |  |
|  | 1.3 Help to move or transfer (0-4 points) | 1.3.1 Relies on trolley for transfer (4 points) |  |  |
|  |  | 1.3.2 Need to rely on wheelchair transfer (3 points) |  |  |
|  |  | 1.3.3 Need to rely on a cane or support to walk (2 points) |  |  |
|  |  | 1.3.4 The patient can walk or move without help, but needs to be reminded or supervised safely (1 point) |  |  |
|  | 1.4 Maintain a comfortable position (0-3 points) | 1.4.1 The patient needs physical restraint due to the need of hemodialysis treatment (3 points) |  |  |
|  |  | 1.4.2 The patient is forced to adopt a position due to the need for hemodialysis treatment or to improve pain (2 points) |  |  |
|  |  | 1.4.3 The patient has no consciousness to change the position at regular time, and needs to be assisted to change the position (1 point) |  |  |
|  | 1.5 Excretion care (0-4 points) | 1.5.1 The patient needs to be discharged through a catheter or fistula (4 points) |  |  |
|  |  | 1.5.2 The patient has normal excretion but needs to use a pad or diaper due to urinary incontinence or fecal incontinence (3 points) |  |  |
|  |  | 1.5.3 The patient's normal excretion is in a suitable container (toilet or chamber pot) and needs assistance (2 points) |  |  |
|  |  | 1.5.4 The patient can get out of bed to go to the toilet, but needs help and supervision (1 point) |  |  |
|  | 1.6 Choose the right clothes (0-2 points) | 1.6.1 Exposure of the limb or hemodialysis catheter on the side of the arteriovenous fistula due to clothing selected by the patient (2 points) |  |  |
|  |  | 1.6.2 The clothing selected by the patient does not affect the exposure of the limb on the side of the arteriovenous fistula or the hemodialysis catheter, but the dressing action cannot be completed by himself (1 point) |  |  |
| 2. Hemodialysis (5-24 points) | 2.1 Monitoring of vital signs (1-4 points) | 2.1.1 The interval time of vital sign monitoring in hemodialysis treatment is less than 15min (4 points) |  |  |
|  |  | 2.1.2 15min ≤ interval time of vital sign monitoring during hemodialysis treatment <30min (3 points) |  |  |
|  |  | 2.1.3 30min ≤ interval time of vital sign monitoring during hemodialysis treatment <60min (2 points) |  |  |
|  |  | 2.1.4 The interval time of vital sign monitoring in hemodialysis treatment =60min (1 minute) |  |  |
|  | 2.2 Invasive nursing operations (e.g., connection and disconnection of hemodialysis catheter and hemodialysis machine, placement of end puncture needle, etc.) (1-3 points) | 2.2.1 The nurse needs to perform three or more invasive operations during dialysis (3 points) |  |  |
|  |  | 2.2.2 The nurse performed two invasive operations during dialysis (2 points) |  |  |
|  |  | 2.2.3 The nurse performed an invasive operation during dialysis (1 point) |  |  |
|  | 2.3 Nursing of the puncture point of internal fistula or blood dialysis catheter (1-3 points) | 2.3.1 During hemodialysis, to avoid continuous exudation of blood or exudate around the puncture point of the fistula or around the catheter, disinfect or cover the skin around the puncture point of the fistula or around the catheter with sterile operation at least once (3 points) |  |  |
|  |  | 2.3.2 During hemodialysis, to promote the healing of the internal fistula puncture after needle removal, gauze or cotton ball was used to apply pressure and bandage the puncture point of the internal fistula for 3 times or more, and the technique used was clean operation (2 points) |  |  |
|  |  | 2.3.3 During hemodialysis, to promote the healing of the internal fistula puncture after needle removal or to maintain the cleanliness of the catheter opening, gauze or cotton ball was used for covering or bandaging 1-2 times, and the technique used was clean operation (1 point) |  |  |
|  | 2.4 Monitoring of intake or weight gain (1-2 points) | 2.4.1 The total ultrafiltration amount or ultrafiltration rate of hemodialysis treatment should be determined by monitoring the liquid inflow and outflow (2 points) |  |  |
|  |  | 2.4.2 Weighing is required to determine the total ultrafiltration volume of hemodialysis treatment (1 point) |  |  |
|  | 2.5 Management of drug therapy (0-3 points) | 2.5.1 In hemodialysis treatment, there are more than one drugs that need strict control of the speed (3 points) |  |  |
|  |  | 2.5.2 Use of three or more drugs in hemodialysis treatment without strict control of the speed (2 points) |  |  |
|  |  | 2.5.3 Use of 1 to 2 drugs in hemodialysis treatment without strict control of the speed (1 point) |  |  |
|  | 2.6 Adjustment of original hemodialysis prescription (prescription content includes: conductivity, temperature, total ultrafiltration, dialysis mode, anticoagulant dose, replacement fluid dose, etc.) (0-3 points) | 2.6.1 Original prescription changed 8 times or more due to various reasons such as blood pressure change, spasm, allergy, elevated pressure of extracorporeal circulation pipeline, bleeding, etc. (3 points) |  |  |
|  |  | 2.6.2 The original prescription was changed 5 to 7 times due to various reasons such as blood pressure change, spasm, allergy, elevated pressure of extracorporeal circulation pipeline, bleeding, etc. (2 points) |  |  |
|  |  | 2.6.3 Original prescription changed 1 to 4 times due to various reasons such as blood pressure change, spasm, allergy, elevated pressure of extracorporeal circulation pipeline, bleeding, etc. (1 point) |  |  |
|  | 2.7 Complications of vascular access (1-4 points) | 2.7.1 The fistula is difficult to puncture and requires an experienced nurse to puncture or the catheter needs thrombolysis (4 points) |  |  |
|  |  | 2.7.2 There are complications of internal fistula (hematoma, collapse, insufficient blood flow, infection, etc.) or complications of catheter (insufficient blood flow, catheter rupture, thrombosis, infection, etc.) (3 points) |  |  |
|  |  | 2.7.3 The method of internal fistula puncture was eye-piercing or catheter without complications (2 points) |  |  |
|  |  | 2.7.4 No complications of internal fistula and rope ladder puncture (1 point) |  |  |
|  | 2.8 Risk monitoring (0-2 points) | 2.8.1 The patient has had previous accidents (catheter slippage, needle slippage, fall, fall from bed, loss of consciousness, etc.) (2 points) |  |  |
|  |  | 2.8.2 The patient has no previous accident, but has high risk factors (physical or cognitive limitations, age over 70 years old, etc.) (1 point) |  |  |
| 3. Social support (0-7 points) | 3.1 Decision support (0-3 points) | 3.1.1 Failure to actively seek help when complications or adverse reactions occur (3 points) |  |  |
|  |  | 3.1.2 Actively seek help from medical staff when complications or adverse reactions occur, but cannot completely follow the guidance of medical staff (2 points) |  |  |
|  |  | 3.1.3 Actively seek help from medical staff when complications or adverse reactions occur, and can follow the guidance of medical staff (1 point) |  |  |
|  | 3.2 Emotional support (0-3 points) | 3.2.1 Have suicidal behavior or suicidal tendency (3 points) |  |  |
|  |  | 3.2.2 There are anxiety, tension, depression, sadness, pain, depression and other emotions (2 points) |  |  |
|  |  | 3.2.3 Insult or quarrel with family members or medical staff (1 point) |  |  |
|  | 3.3 Material support (0-1 point) | 3.3.1 The patient's family is unwilling to provide more material and economic help for the treatment of the disease (1 point) |  |  |

Scale Development Information

Scale Title: Hemodialysis Patient Care Dependency Scale (HD-CDS)

Developers: Cong Liang, Hongyan Niu, and Lina Xue

Year of Development: 2024

Licensing: This scale is made available under the Creative Commons Attribution 4.0 International (CC BY 4.0) license. Users are free to share and adapt the scale, provided that appropriate credit is given to the original authors.
