# Supplementary material for: Development and preliminary psychometric evaluation of the hemodialysis patient care dependency scale (HD-CDS)
Source: PLoS One. 2026 Sep 15;21(9):e0358118. doi: 10.1371/journal.pone.0358118 (PMC13577502; doi:10.1371/journal.pone.0358118)
Supplement: Supplementary file 3 [file pone.0358118.s007.docx]

**Development and Psychometric Evaluation of the Hemodialysis Patient Care Dependency Scale(HD-CDS) Based on the Factor System Scoring Method**

**PLOS ONE Peer review**

**1. Technical Soundness and Data Support**

**The manuscript is technically solid.** The authors employed an exploratory sequential mixed-methods design, which is the gold standard for scale development.

**Phase 1 (Qualitative):** The use of the Nominal Group Technique (NGT) and a two-round Delphi process with 18 experts ensures that items are grounded in both clinical expertise and Henderson's Theory of Basic Human Needs.

**Phase 2 (Quantitative):** With a sample size of 395 for a 17-item scale, the item-to-participant ratio is strong for robust Exploratory Factor Analysis (EFA).

**Conclusions:** The data support the authors’ claims. The internal consistency is high (Cronbach’s α = 0.893), and test-retest reliability is excellent (ICC = 0.965). EFA produced a three-factor structure explaining 69.68% of the variance, which is exceptional for nursing assessment tools, indicating that the scale effectively captures the intended constructs.

**2. Statistical Analysis**

The statistical methods are **appropriate and rigorous** for psychometric validation:

**Item Analysis:** The critical ratio method and corrected item-total correlation (CITC) ensure that only discriminative items were retained.

**Validity:** Construct validity was confirmed via EFA with Varimax rotation. Criterion validity was established by correlating HD-CDS with the Barthel Index (r = −0.715, P < 0.01). This negative correlation is logically consistent, as higher dependency (HD-CDS) aligns with lower self-care ability (Barthel Index).

**Reliability:** Multiple reliability measures—including Cronbach’s alpha, split-half, and ICC—provide a comprehensive view of the scale’s performance.

**3. Data Availability**

There is a contradiction in the manuscript regarding data availability that must be resolved for PLOS ONE compliance:

- Pages 6–7: Data are stated as fully available within the manuscript or supporting files.
- Page 29: Data are said to be available on request from the corresponding author.

**Recommendation:** The authors should provide the full anonymized dataset (e.g., .csv or .xlsx) as a Supporting Information file to meet PLOS ONE’s open data requirements.

**4. Presentation and English**

The manuscript is written in **high-quality academic English: clear, concise, and logically structured.**

**Minor formatting issues:**

- Page 24: "Figure 1.scree plot" lacks a space.
- Page 9: Full-width punctuation (commas and colons) should be converted to standard English.

**5. Additional Comments**

**Major discrepancy in study dates:**

Page 11 (Methods): "August 2024 to December 2024."

Page 15 (Participants): "01/12/2022 to 01/10/2024."

Clarification is needed on whether the August–December 2024 period refers only to the Delphi process or statistical analysis, as the participant recruitment dates suggest that the quantitative phase began much earlier.

**Reviewed by:** Abdelrahim Dafalla Elhag Ahmed
