# Supplementary material for: Development and preliminary psychometric evaluation of the hemodialysis patient care dependency scale (HD-CDS)
Source: PLoS One. 2026 Sep 15;21(9):e0358118. doi: 10.1371/journal.pone.0358118 (PMC13577502; doi:10.1371/journal.pone.0358118)
Supplement: Supplementary file 4 [file pone.0358118.s008.docx]

It’s a priviledge reviewing this manuscript titled: Development and Psychometric Evaluation of the Hemodialysis Patient Care Dependency Scale(HD-CDS) Based on the Factor System Scoring Method.

This is a relevant and important topic in view of the global burden of chronic kidney disease and the many challenges faced by the affected patients. Below are my comments.

1. Although the authors made reference to the Henderson theory, they did not conceptualise the term ‘care dependency used in this study’ adequately. There is a need to provide a stronger and sufficient conceptual framework that will show how care dependency is different from frailty and functional status and relate it to the final three domains that were derived at the end of the study.
2. The authors said, ‘This study was conducted from August 2024 to December 2024 using….’ Under the study design section of the methods. They also said, ‘Patient recruitment and data collection spanned from 01/12/2022 to 01/10/2024.’ They need to clarify these discrepancies.
3. The authors said ‘…..Each expert rated each item from 1 (not relevant) to 4 (very relevant and concise). The item-level content validity index (I-CVI) is calculated as the number of experts who scored the item as 1 divided by the total number of experts….’ I think there is something wrong with the method by which it was calculated. The standard method to calculate the I-CVI for a single item is to first dichotomise the expert ratings on the Likert scale from 1 to 4. Ratings of 3 and 4 are considered "content valid" (agreement), while ratings of 1 and 2 are considered "not valid." The formula is: The number of experts who gave a rating of 3 or 4 divided by the total number of experts in the panel. The authors need to clarify the statement, correct the computation method and possibly report the corrected values.
4. Despite being statistically significant, the reported Kendall's W values (e.g., 0.148–0.235) were low, meaning that there is limited agreement. The assertion of "high consensus" is refuted by this. It is recommended that the authors be extra cautious when interpreting Kendall's W. They should also discuss how low agreement affects content validity and the process that was used to settle these disagreements.

Above all, this is a good manuscript that is worth publishing; the above methodological and reporting issues need to be corrected before this can be done.
