# Supplementary material for: Development and preliminary psychometric evaluation of the hemodialysis patient care dependency scale (HD-CDS)
Source: PLoS One. 2026 Sep 15;21(9):e0358118. doi: 10.1371/journal.pone.0358118 (PMC13577502; doi:10.1371/journal.pone.0358118)
Supplement: Supplementary file 5 [file pone.0358118.s010.docx]

Response to Journal Requirements：

**Journal Requirement 1:** Please ensure that your manuscript meets PLOS ONE's style requirements, including those for file naming. The PLOS ONE style templates can be found at <https://journals.plos.org/plosone/s/file?id=wjVg/PLOSOne_formatting_sample_main_body.pdf> and <https://journals.plos.org/plosone/s/file?id=ba62/PLOSOne_formatting_sample_title_authors_affiliations.pdf>.

**Response:** Thank you for this reminder. We have carefully checked and revised the manuscript according to the PLOS ONE style requirements and formatting templates, including the title page, main text structure, references, tables/figures, and file naming. In addition, we requested professional English editing and journal-specific formatting support from LetPub to ensure that the revised manuscript conforms to the requirements of PLOS ONE. The revised clean manuscript and the marked-up manuscript have been prepared and named according to the journal’s submission requirements.

**Journal Requirement 2:** Thank you for stating the following financial disclosure: “Science and Technology Project for the Development and Enhancement of Nursing Discipline at The First People's Hospital of Changzhou, yy2023001 and yy2023015.”

Please state what role the funders took in the study. If the funders had no role, please state: “The funders had no role in study design, data collection and analysis, decision to publish, or preparation of the manuscript.” If this statement is not correct you must amend it as needed.

Please include this amended Role of Funder statement in your cover letter; we will change the online submission form on your behalf.

**Response:** Thank you for your reminder. We have included the amended Funding and Role of Funder Statement in the cover letter as requested. In addition to the Science and Technology Project for the Development and Enhancement of Nursing Discipline at The First People's Hospital of Changzhou, yy2023001 and yy2023015, we have added the Changzhou Municipal Health Commission Young Talent Science and Technology Project (No. QN202443), which supported only the professional English language editing of the revised manuscript. This funding source was added during revision because professional native English editing was requested during the review process. The funders had no role in study design, data collection and analysis, interpretation of results, or decision to publish. The Changzhou Municipal Health Commission Young Talent Science and Technology Project (No. QN202443) had no role other than supporting the professional English language editing of the revised manuscript.

**Response:** Thank you for your reminder. We have included the amended Role of Funder statement in the cover letter as requested. The statement is as follows: “This study was supported by the Science and Technology Project for the Development and Enhancement of Nursing Discipline at The First People's Hospital of Changzhou, yy2023001 and yy2023015. The funders had no role in study design, data collection and analysis, decision to publish, or preparation of the manuscript.” In accordance with the journal’s requirements, funding-related text has been removed from the manuscript and will only be provided in the cover letter and the Funding Statement section of the online submission form.

**Journal Requirement 3:** We note that your Data Availability Statement is currently as follows: “All relevant data are within the manuscript and its Supporting Information files.” Please confirm at this time whether or not your submission contains all raw data required to replicate the results of your study. Authors must share the “minimal data set” for their submission.

**Response:** Thank you for your reminder. We confirm that our submission contains all anonymized raw data required to replicate the findings reported in this study. The minimal data set has been uploaded as Supporting Information files, including S1–S4 Excel files. Specifically, S1_Data contains participant demographic and clinical characteristics, item scores for item analysis and reliability/validity analyses, and criterion validity (Barthel Index) data; S2_Data contains the raw ratings for content validity (I-CVI); S3_Data contains the test-retest scores for ICC analysis; and S4_Data contains the detailed statistical output for the analyses summarized in Table 5. We have revised the Data Availability Statement accordingly. The revised statement can be found in the Revised Manuscript with Tracked Changes and Professional English Editing, Page 23, Lines 21–27.

**Journal Requirement 4:** Please be informed that funding information should not appear in the Acknowledgments section or other areas of your manuscript. We will only publish funding information present in the Funding Statement section of the online submission form. Please remove any funding-related text from the manuscript.

**Response:** Thank you for your clarification. We have removed all funding-related text from the manuscript, including the Acknowledgments section and any other areas of the manuscript. The funding information and the Role of Funder statement have been provided in the cover letter as requested and will be included in the Funding Statement section of the online submission form.The funding statement originally located on Page 23 has been removed.

**Journal Requirement 5:** Your ethics statement should only appear in the Methods section of your manuscript. If your ethics statement is written in any section besides the Methods, please delete it from any other section.

**Response:** Thank you for your reminder. We have carefully checked the manuscript and ensured that the ethics statement appears only in the Methods section. The retained ethics statement is presented under the subsection “Ethical considerations” in the Revised Manuscript with Tracked Changes, Page 9, Lines 25–31, and Page 10, Lines 1–2. The duplicate ethics statement previously included outside the Methods section at the end of the manuscript has been removed. The deletion can be found in the Revised Manuscript with Tracked Changes, Page 23.

**Journal Requirement 6:** If the reviewer comments include a recommendation to cite specific previously published works, please review and evaluate these publications to determine whether they are relevant and should be cited. There is no requirement to cite these works unless the editor has indicated otherwise.

**Response:** Thank you for this clarification. We have carefully reviewed the reviewer comments and confirm that no reviewer requested or recommended the citation of specific previously published works. Therefore, no additional references were added in response to this requirement.

**Additional Editor Comment 1:** Please mention in the method how many participants in this research.

**Response:** Thank you for your suggestion. We have clarified the number of participants in the revised manuscript. The number of participants is mentioned in the Abstract, Page 1, Line 15, and further described in the Methods section, Page 8, Line 12. Specifically, the revised manuscript states that 395 participants were included in the psychometric evaluation, and that the retest sample accounted for approximately10% of the total sample (n = 39).

**Additional Editor Comment 2:** Please make analysis between sociodemographic and the scoring.

**Response:** Thank you for your suggestion. We have added analyses examining the associations between sociodemographic/clinical variables and HD-CDS total scores. The statistical methods used for these analyses have been described in the Statistical analysis section of the Revised Manuscript with Tracked Changes, Page 9, Lines 19–24. The results are presented in the Results section under “Correlates of HD-CDS scores” and in Table 5, both of which can be found in the Revised Manuscript with Tracked Changes, Page 18.

**Additional Editor Comment 3:** Explain in the discussion about correlation sociodemographic and the scoring.

**Response:** Thank you for your suggestion. We have added an explanation in the Discussion section regarding the relationships between sociodemographic/clinical variables and HD-CDS scores. We discussed that patients with lower education levels, more frequent HD sessions, and temporary or more complex vascular access types tended to have higher care dependency scores. We also explained the negative correlations of dialysis vintage and pre-dialysis blood pressure with care dependency, and noted that sex, age, and ultrafiltration rate were not significantly associated with HD-CDS scores. This explanation can be found in the Revised Manuscript with Tracked Changes, Page 19, Line 26 to Page 20, Line 9.

**Response to Reviewer Comments**

We sincerely thank the reviewer for the positive evaluation of our manuscript and for recognizing the value of this study. We are grateful for the reviewer’s constructive comments and suggestions, which have helped us further improve the clarity, rigor, and overall quality of the manuscript. We have carefully addressed each comment and revised the manuscript accordingly. Detailed point-by-point responses are provided below.

**Reviewer Comment 1:** The manuscript needs serious professional editing. Examples: “may be crucial” looks too weak; “was also accepted” feels awkward; “we conceptualize” has tense issue; “assessment work better guarantees” has incorrect grammar. Additionally, there are many spacing/punctuation issues. I recommend the authors to use native English editing.

**Response:** Thank you for your valuable comment. We fully agree that the language quality of the manuscript needed substantial improvement. After completing all revisions and additions in response to the editor’s and reviewers’ comments, we submitted the revised manuscript to LetPub for professional English language editing by native English-speaking editors. The manuscript has been thoroughly edited for grammar, wording, tense consistency, punctuation, spacing, and overall readability. The examples mentioned by the reviewer, as well as similar language issues throughout the manuscript, have been corrected. We have also provided the language editing certificate from LetPub as supporting documentation.

**Reviewer Comment 2:** In submission form it says all relevant data are within manuscript and supporting files. But in manuscript it says “Data available from corresponding author on reasonable request”. These contradict each other. The authors should fix this.

**Response:** Thank you for pointing out this inconsistency. We have revised the Data Availability Statement to ensure consistency between the manuscript and the submission form. The previous statement indicating that data were available from the corresponding author upon reasonable request has been removed. The revised manuscript now states that all anonymized raw data required to replicate the findings of this study have been uploaded as Supporting Information files, including S1–S4 Excel files. This correction has also been described in detail in our response to Journal Requirement 3.

**Reviewer Comment 3:** There are some issues with timeline consistency. The Methods says “Study conducted August 2024 to December 2024”. But recruitment says 01/12/2022 to 01/10/2024. Need correction or explanation.

**Response:** Thank you for pointing out this inconsistency. We apologize for this error. The dates reported in the Participants section were incorrect. The correct timeline of the study is as follows: the scale development phase was conducted from August 1, 2024, to September 30, 2024, and the psychometric evaluation phase was conducted from October 1, 2024, to December 1, 2024.

We have revised the Design section to clearly state the two stages and their specific dates: “This study was conducted from August 2024 to December 2024 using an exploratory sequential mixed methods design. The research comprised two stages: (1) scale development (August 1, 2024, to September 30, 2024) and (2) psychometric evaluation (October 1, 2024, to December 1, 2024), which included refining the scale and assessing its psychometric properties.”

We have also corrected the Participants section. The original incorrect statement, “from 01/12/2022 to 01/10/2024,” has been replaced with: “Patient recruitment and data collection spanned from October 1, 2024, to December 1, 2024.” All timeline inconsistencies have therefore been corrected in the revised manuscript. These revisions can be found in the Revised Manuscript with Tracked Changes, Page 3, Lines 24–28.

**Reviewer Comment 4:** The criterion validity needs better justification. You used Barthel Index with correlation = -0.715. It is reasonable, but Barthel measures ADL, not dialysis dependency. Why you used Barthel instead of nursing workload or dependency gold standard? This needs stronger explanation.

**Response:** Thank you for this important comment. We agree that the rationale for using the Barthel Index as the criterion measure needed to be explained more clearly. We have added a new paragraph in the Discussion section to justify this choice. In the revised manuscript, we explain that the Barthel Index is widely used and validated for assessing activities of daily living and has conceptual overlap with care dependency, as patients with lower self-care ability generally require greater nursing assistance. We also acknowledge its limitations, including that it was not specifically designed to measure care dependency and does not capture dialysis-specific nursing needs. However, in the absence of an established gold standard for assessing care dependency among patients undergoing hemodialysis, we considered the Barthel Index to be a reasonable proxy criterion for evaluating criterion-related validity. This explanation can be found in the Revised Manuscript with Tracked Changes, Page 19, Lines 16–25.

**Reviewer Comment 5:** Some Statistical Descriptions Are Weak. Examples: “ICC was accepted”, and “good discriminative ability”. It needs confidence intervals for alpha if possible. And also need clearer rationale for retaining Item 3.3 despite low correlation.

**Response:** Thank you for these valuable suggestions. We have strengthened the statistical descriptions in the revised manuscript.

First, we added 95% confidence intervals for Cronbach’s α for the overall scale and each dimension. The revised manuscript now reports that the Cronbach’s α coefficient for the overall scale was 0.893 (95% CI: 0.877–0.908), and the coefficients for the three dimensions were 0.921 (95% CI: 0.908–0.932), 0.878 (95% CI: 0.859–0.895), and 0.737 (95% CI: 0.689–0.779), respectively. We also provided confidence intervals for split-half reliability.

Second, we revised the description of test–retest reliability and replaced the previous vague wording with a clearer criterion. In the Reliability analysis section, we now state that an ICC value greater than 0.75 was considered acceptable for test–retest reliability. The obtained ICC of 0.965 exceeded this threshold, supporting the excellent stability of the scale.

Third, we added a detailed explanation for retaining Item 3.3 despite its relatively low corrected item-total correlation. Item 3.3 showed a corrected item-total correlation of 0.313. However, it was retained for two reasons. First, qualitative findings from the nominal group technique and expert panel discussions indicated that this item was clinically important, because insufficient material or financial support from the patient’s family may increase care dependency and require nurses to compensate for unmet support needs. Second, the item’s score range was only 0–1 point, which limited its influence on the overall scale variance, and its correlation with the total score remained statistically significant (P < 0.01). Therefore, we retained Item 3.3 based on both clinical relevance and statistical significance.

The revisions regarding confidence intervals and reliability descriptions can be found in the Revised Manuscript with Tracked Changes, Page 17, Line 11 to Page 18, Line 9. The rationale for retaining Item 3.3 can be found in the Revised Manuscript with Tracked Changes, Page 14, Line 22 to Page 15, Line 3.

**Reviewer Comment 6:** Ethical Statement Needs Minor Editing. Use: “Written informed consent was obtained from all participants.” Not “consents”.

**Response:** Thank you for pointing this out. As requested in Journal Requirement 5, the ethics statement should appear only in the Methods section. Therefore, the duplicate ethics statement at the end of the manuscript, where this wording issue originally appeared, has been removed. The retained ethics statement now appears only in the Methods section under “Ethical considerations,” and the wording has been checked to ensure that “Written informed consent was obtained from all participants” is used appropriately.

**Reviewer Comment 7:** All patients are from one hospital in Changzhou. This limits generalizability. So, it needs stronger limitation statement.

**Response:** Thank you for this important comment. We agree that the single-center design limits the generalizability of the findings. We have strengthened the limitation statement in the revised manuscript to explicitly acknowledge that all participants were recruited from one hospital in Changzhou and that this may restrict the applicability of the findings to other regions, institutions, or broader hemodialysis populations. This limitation is described in the Limitations section of the Revised Manuscript with Tracked Changes, Page 21, Lines 29–33.

**Reviewer Comment 8:** Who exactly completed HD-CDS? Sometimes says nurses scored it. Sometimes patients participated. This needs clarity. Is it observer-rated? Nurse-administered? Patient interview?

**Response:** Thank you for this important clarification. We have revised the Methods section, under “Data collection,” to clarify who completed the HD-CDS. The revised manuscript states: “The HD-CDS was completed by primary nurses who had been trained on the scale content. These nurses evaluated patients who met the inclusion and exclusion criteria, and the assessment continued until all items of the scale were fully completed.” This clarification can be found in the Revised Manuscript with Tracked Changes, Page 8, Lines 18–21.

**Reviewer Comment 9:** The current title is too long. Better: “Development and Psychometric Validation of the Hemodialysis Patient Care Dependency Scale (HD-CDS).”

**Response:** Thank you for this helpful suggestion. We agree that the original title was too long. We have revised the title from “Development and Psychometric Evaluation of the Hemodialysis Patient Care Dependency Scale (HD-CDS) Based on the Factor System Scoring Method” to “Development and psychometric validation of the hemodialysis patient care dependency scale (HD-CDS).”

**Revisions and Responses to Additional Comments Included in the Attached Review Files**

**Additional Comment:** The manuscript needs serious professional editing. Page 24: “Figure 1.scree plot” lacks a space. Page 9: Full-width punctuation should be converted to standard English.

**Response:** Thank you for these helpful comments. After completing the revisions, we submitted the manuscript to LetPub for professional English language editing. The manuscript has been carefully edited for grammar, wording, punctuation, spacing, and overall readability. In addition, we have corrected the specific formatting issues mentioned by the reviewer, including the missing space in “Figure 1. scree plot” and the use of full-width punctuation.

**Additional Comment:** Although the authors made reference to the Henderson theory, they did not conceptualise the term “care dependency” used in this study adequately. There is a need to provide a stronger and sufficient conceptual framework that will show how care dependency is different from frailty and functional status and relate it to the final three domains that were derived at the end of the study.

**Response:** Thank you for this important comment. We have strengthened the conceptual framework of the manuscript. First, we added an operational definition of care dependency in patients undergoing hemodialysis and distinguished it from frailty and functional status in the Introduction. This revision can be found in the Revised Manuscript with Tracked Changes, Page 3, Lines 11–16. Second, we clarified that the three-factor structure was hypothesized based on Henderson’s theory and qualitative findings and was then tested by exploratory factor analysis. Third, we further explained in the Discussion why the three dimensions are specific to patients undergoing hemodialysis. This explanation can be found in the Revised Manuscript with Tracked Changes, Page 19, Lines 9–12. We believe these revisions have substantially strengthened the conceptual framework of the study.

**Additional Comment:** The item-level content validity index (I-CVI) is calculated as the number of experts who scored the item as 1 divided by the total number of experts. The standard method is that ratings of 3 and 4 are considered content valid.

**Response:** Thank you for pointing this out. We apologize for the inaccurate description of the I-CVI calculation in the Methods section. We would like to clarify that the actual I-CVI values reported in the manuscript were calculated correctly, using the standard method: the number of experts rating an item as 3 or 4 divided by the total number of experts. Therefore, the calculation results were not affected; the error was limited to the written description of the statistical method. The original data for I-CVI have been provided for verification. We have corrected the description in the revised manuscript. This correction can be found in the Revised Manuscript with Tracked Changes, Page 6, Line 25.

**Additional Comment:** Despite being statistically significant, the reported Kendall’s W values were low. The assertion of “high consensus” is refuted by this. Authors should be cautious when interpreting Kendall’s W and discuss how low agreement affects content validity and the process used to settle disagreements.

**Response:** Thank you for this important correction. We apologize for the inappropriate interpretation of the Kendall’s W coefficients in the original manuscript. We agree that statistically significant Kendall’s W values do not necessarily indicate high consensus. Therefore, we revised the Results section to describe these findings more cautiously. The revised manuscript now states: “Although significant (all p < 0.05), the relatively low Kendall’s W values for the first- and second-level items (ranging from 0.148 to 0.235) indicate only fair to moderate agreement among experts. The third-level items showed higher agreement (0.689 in round 1 and 0.290 in round 2).” This revision can be found in the Revised Manuscript with Tracked Changes, Page 11, Lines 2–5.
