# Supplementary material for: Development and preliminary psychometric evaluation of the hemodialysis patient care dependency scale (HD-CDS)
Source: PLoS One. 2026 Sep 15;21(9):e0358118. doi: 10.1371/journal.pone.0358118 (PMC13577502; doi:10.1371/journal.pone.0358118)
Supplement: Supplementary file 6 [file pone.0358118.s011.docx]

**Response to the Academic Editor and Reviewers**

**Manuscript ID:** PONE-D-25-67462R1

**Manuscript title:** Development and preliminary psychometric evaluation of the hemodialysis patient care dependency scale (HD-CDS)

Dear Dr. Kaewput and Reviewers,
Thank you for the careful evaluation of our revised manuscript and for the constructive guidance. We have addressed each point below. The comments are reproduced in full, followed by our responses and the corresponding manuscript locations.

**Response to Journal Requirements**

**Journal Requirement 1:** If the reviewer comments include a recommendation to cite specific previously published works, please review and evaluate these publications to determine whether they are relevant and should be cited. There is no requirement to cite these works unless the editor has indicated otherwise.

**Response:** No reviewer requested citation of a specific work in this round. We therefore did not add citations solely in response to a reviewer recommendation.

**Journal Requirement 2:** Please review your reference list to ensure that it is complete and correct. If you have cited papers that have been retracted, please include the rationale for doing so in the manuscript text, or remove these references and replace them with relevant current references. Any changes to the reference list should be mentioned in the rebuttal letter that accompanies your revised manuscript. If you need to cite a retracted article, indicate the article’s retracted status in the References list and also include a citation and full reference for the retraction notice.

**Response:** We reviewed the reference list for completeness, bibliographic consistency, and retraction status. No cited item was identified as retracted. Minor hyperlink and formatting corrections were made without adding or removing any reference; the reference numbering is unchanged.

**Response to Additional Editor Comments**

**Additional Editor Comment 1:** Please verify and report the test–retest sample size consistently throughout the manuscript, abstract, response letter, tables, and Supporting Information. Different sections appear to report either 39 or 40 participants. If 40 participants were invited but only 39 were included in the analysis, please state this explicitly and briefly explain the exclusion or missing assessment.

**Response:** Thank you for identifying this inconsistency. Forty participants completed the two-week retest. During data verification, we found that one participant had experienced an acute gastrointestinal bleeding event during the retest interval. This clinically important change in health status violated the stability assumption for test–retest reliability, so this participant was excluded from the reliability analysis. The final test–retest analysis therefore included 39 clinically stable participants. We now report this distinction consistently in the Abstract, Methods, Results, and S3 Data. S3 Data contains 39 analyzed participants.

**Location of changes:** Abstract (page 1); Methods—Participants and Data collection (pages 8–9); Methods—Statistical analysis (page 9); Results—Reliability analysis (page 18); S3 Data.

**Revised manuscript text:** *Forty participants completed a second HD-CDS assessment two weeks after the initial assessment. During data verification, one participant was found to have experienced an acute gastrointestinal bleeding event during the retest interval. Because this represented a clinically important change in health status and violated the stability assumption underlying test–retest reliability assessment, this participant was excluded. Therefore, 39 participants were included in the test–retest analysis.*

**Additional Editor Comment 2:** The ethics statement in the submission information still states, “Written informed consents were obtained from all participants.” Please revise this to, “Written informed consent was obtained from all participants.” Please also ensure that the ethics statement entered in the submission system is identical to the statement in the Methods section.

**Response:** We corrected the ethics statement in the Methods section to the requested singular construction. At resubmission, we will also enter the identical sentence in the submission system so that the online statement and manuscript match exactly.

**Location of changes:** Methods—Ethical considerations (page 10); submission-system ethics field at resubmission.

**Revised manuscript text:** *Written informed consent was obtained from all participants.*

**Additional Editor Comment 3:** Because the Barthel Index is not a gold-standard measure of hemodialysis-specific care dependency, the observed correlation may be more accurately described as evidence of convergent validity rather than definitive criterion validity. Please either replace “criterion validity” with “convergent validity” throughout the Abstract, Methods, Results, Discussion, tables, and Supporting Information, or consistently describe the Barthel Index as a proxy criterion and clearly acknowledge the limitations of this terminology.

**Response:** We adopted the preferred terminology of convergent validity throughout the manuscript. We now explain that the Barthel Index measures activities of daily living and is conceptually related to care dependency but is not a gold-standard measure of hemodialysis-specific care dependency. We also prespecified the expected negative direction because higher HD-CDS scores indicate greater care dependency whereas higher Barthel Index scores indicate greater functional independence.

**Location of changes:** Abstract (page 1); Methods—Convergent validity (pages 7–8); Results—Convergent validity (page 18); Discussion (page 20); Availability of data and materials (page 23).

**Revised manuscript text:** *Convergent validity was examined using Pearson’s correlation. The Barthel Index[27] was selected as the comparator because it assesses functional independence in activities of daily living, a construct conceptually related to care dependency. However, it does not directly assess hemodialysis-specific care needs. A negative correlation was hypothesized because higher HD-CDS scores indicate greater care dependency, whereas higher Barthel Index scores indicate greater functional independence.*

**Additional Editor Comment 4:** Some statements remain stronger than the available evidence supports, including descriptions of the HD-CDS as a “reliable and effective” or fully “validated” instrument and suggestions that it can already guide targeted nursing interventions or staffing allocation. The present study provides promising initial evidence of content validity, internal consistency, test–retest reliability, and exploratory structural validity, but it does not directly evaluate staffing decisions, nursing workload, safety outcomes, or clinical effectiveness. Please consider revising the conclusion as follows: “The HD-CDS demonstrated promising preliminary psychometric properties in a single-center sample and may provide a useful framework for assessing care dependency among patients undergoing hemodialysis. Further multicenter studies, including independent structural validation and inter-rater reliability assessment, are required before routine clinical implementation.” Similarly, please remove or qualify claims that the instrument has “superior” assessment capabilities or can reduce falls, infections, or other safety events unless these outcomes were directly evaluated.

**Response:** We agree and revised the title, Abstract, Discussion, Limitations, Conclusion, and Supporting Information description to distinguish an initial psychometric evaluation from external clinical validation. We removed or qualified claims that the HD-CDS is fully validated, reliable and effective, superior, able to direct staffing, or able to reduce falls, infections, or other safety events. The Discussion now states explicitly that nursing workload, staffing allocation, clinical effectiveness, and patient-safety outcomes were not evaluated. Potential uses in care planning and resource allocation are presented as hypotheses for future study. The Conclusion uses the editor’s recommended cautious formulation.

**Location of changes:** Title and Abstract (pages 1–2); Discussion (pages 19–21); Limitations (pages 21–22); Conclusion (page 22); Supplementary Information description (page 23).

**Revised manuscript text:** *The HD-CDS may provide a structured framework for describing care dependency across basic needs, HD treatment, and social support. However, the present study did not evaluate nursing workload, staffing allocation, clinical effectiveness, falls, infections, or other patient-safety outcomes. Therefore, its potential applications in individualized care planning and resource allocation should be regarded as hypotheses for future evaluation rather than established clinical benefits.*

**Revised manuscript text:** *The HD-CDS demonstrated promising preliminary psychometric properties in a single-center sample and may provide a useful framework for assessing care dependency among patients undergoing hemodialysis. Further multicenter studies, including independent structural validation and inter-rater reliability assessment, are required before routine clinical implementation.*

**Additional Editor Comment 5:** Please identify the specific factor-extraction method used rather than reporting only the rotation method. In addition, the manuscript states that the loading threshold of 0.40 was calculated using 5.152/√(n−2). For n = 395, this calculation gives approximately 0.26 rather than 0.40. Please correct this statement. If 0.40 was selected as an a priori practical threshold, please state this directly and remove the inconsistent calculation.

**Response:** We now identify principal component analysis as the extraction method and orthogonal Varimax as the rotation method in the Methods, Results, and Table 4 title. We removed the inconsistent 5.152/√(n−2) calculation and state directly that a loading of at least 0.40 was selected a priori as a practical threshold for interpreting salient loadings. The statistical software remains reported as SPSS for Mac, version 27.0.1.

**Location of changes:** Methods—Construct validity (page 7); Methods—Statistical analysis (page 9); Results—Exploratory factor analysis and Table 4 (pages 16–17).

**Revised manuscript text:** *Principal component analysis was used for factor extraction, followed by orthogonal Varimax rotation. … A factor loading of ≥ 0.40 was selected a priori as a practical threshold for interpreting salient loadings[25].*

**Additional Editor Comment 6:** The social-support domain had a Cronbach’s α of 0.737 and a split-half coefficient of 0.755, whereas the Methods specify values greater than 0.80 as acceptable. Please reconcile this inconsistency. The social-support domain may be described as showing moderate or acceptable preliminary internal consistency, but the manuscript should not state that every dimension exceeded the prespecified threshold. The overall scale reliability remains satisfactory.

**Response:** We retained the prespecified threshold and revised the interpretation so that the results are transparent. The Results now state that the overall scale and the basic-needs and HD-treatment dimensions showed satisfactory internal consistency, whereas the social-support dimension did not meet the prespecified > 0.80 threshold and showed moderate preliminary internal consistency. We also added a caution in the Discussion that the lower social-support coefficients require examination in an independent sample.

**Location of changes:** Results—Reliability analysis (page 18); Discussion (page 19).

**Revised manuscript text:** *The overall scale and the basic-needs and HD-treatment dimensions showed satisfactory internal consistency. However, the social-support dimension did not meet the prespecified threshold of > 0.80, with a Cronbach’s α of 0.737 and a split-half coefficient of 0.755, indicating moderate preliminary internal consistency.*

**Revised manuscript text:** *Although the overall scale showed satisfactory internal consistency, the lower coefficients for the social-support dimension warrant further examination in an independent sample.*

**Additional Editor Comment 7:** Please conduct a final review of the manuscript to ensure that very small p values are reported as “p < 0.001” rather than “p = 0.000,” the domain names are used consistently throughout, duplicated or tracked-change text is removed, the clean manuscript contains no editorial comments, and the distinction between initial psychometric evaluation and external clinical validation is maintained consistently.

**Response:** We completed a final review of the manuscript and Supporting Information. Very small p values are now reported as p < 0.001 in the text, tables, and S4 Data. The domain names are standardized as basic needs, HD treatment, and social support, including in S2 Data. The clean manuscript contains no tracked changes or editorial comments. Duplicate text was removed, and language throughout the title, Abstract, Discussion, Limitations, Conclusion, and Supporting Information consistently distinguishes promising preliminary psychometric evidence from independent structural validation, inter-rater reliability, and external clinical validation.

**Location of changes:** Throughout the manuscript; Tables 2–5; S2 Data and S4 Data; with particular emphasis on the title and Abstract (pages 1–2), Discussion and Limitations (pages 19–22), and Conclusion (page 22).

**Response to Reviewer #2**

**Reviewer #2 Comment:** The authors have addressed the earlier concerns; however, the limitation section would benefit from further elaboration, particularly regarding the justification of generalizability. The current formulation acknowledges this issue only “to some extent,” which leaves the reader without a clear understanding of how the findings can be applied beyond the study context. Strengthening this part with a more explicit and well-reasoned justification—one that clearly aligns with and supports your final argument—is necessary before the manuscript is ready for publication.

**Response:** Thank you for this important recommendation. We substantially strengthened the Limitations section. It now explains why a single-center sample limits transportability, identifies the contextual factors that may differ across settings, states explicitly that the present findings cannot yet be generalized beyond the study setting, and specifies the multicenter and independent validation evidence needed before broader application. This wording aligns with the revised cautious Conclusion.

**Location of changes:** Limitations (page 21); Conclusion (page 22).

**Revised manuscript text:** *Second, all participants were recruited from a single tertiary hospital in Changzhou, China. The patient case mix, staffing patterns, nurse training, care workflows, and local clinical practices may differ from those in other hospitals, regions, and health-care systems. Therefore, the present findings should be interpreted as preliminary evidence and cannot yet be generalized beyond the study setting. Multicenter studies involving different regions and facility levels, together with independent structural validation, are required to evaluate the external validity and transportability of the HD-CDS.*

**Response to Reviewer #3**

**Reviewer #3 Comment:** Thank you for addressing the reviewers' comments and for the substantial revisions made to the manuscript. The revised version is considerably improved in terms of clarity, methodological reporting, and conceptual framework. You have adequately addressed my major concerns by: Strengthening the conceptualization of care dependency and clearly distinguishing it from frailty and functional status, while linking the concept to the three domains of the HD-CDS. Correcting the inconsistencies in the study timeline. Correcting the description of the item-level content validity index (I-CVI) calculation and clarifying that the analyses were performed using the standard approach. Revising the interpretation of Kendall's W values to avoid overstating expert consensus. I also note that the data availability statement has been revised, and the authors have indicated that the anonymized datasets supporting the findings have been provided as supporting information, in keeping with the journal's requirements. Overall, I believe the manuscript has been substantially improved and is suitable for publication.

**Response:** We sincerely thank Reviewer #3 for the careful reassessment and positive evaluation. No additional revision was requested in this comment. We have maintained the conceptual, methodological, and data-availability clarifications noted by the reviewer while completing the editor-requested minor revisions.

**Response to Reviewer #4**

**Reviewer #4 Comment:** The authors have comprehensively addressed all reviewer feedback, and the manuscript has been significantly improved. I am pleased with the final version and strongly recommend acceptance for publication.

**Response:** We sincerely thank Reviewer #4 for the positive evaluation and recommendation. No additional revision was requested in this comment.

We again thank the Academic Editor and Reviewers for their time and constructive guidance.
