# Supplementary material for: Development and preliminary psychometric evaluation of the hemodialysis patient care dependency scale (HD-CDS)
Source: PLoS One. 2026 Sep 15;21(9):e0358118. doi: 10.1371/journal.pone.0358118 (PMC13577502; doi:10.1371/journal.pone.0358118)
Supplement: Supplementary file 7 [file pone.0358118.s012.docx]

**Response to the Academic Editor**

**Manuscript ID:** PONE-D-25-67462R2

**Manuscript title:** Development and preliminary psychometric evaluation of the hemodialysis patient care dependency scale (HD-CDS)

Dear Dr. Kaewput,
Thank you for the careful evaluation of our revised manuscript and for identifying the remaining editorial and methodological consistency issues. We have addressed each point below. Because the Additional Editor Comments repeat the Academic Editor comments verbatim, each distinct concern is reproduced and answered once in this response.

**Response to Journal Requirements**

**Journal Requirement 1:** If the reviewer comments include a recommendation to cite specific previously published works, please review and evaluate these publications to determine whether they are relevant and should be cited. There is no requirement to cite these works unless the editor has indicated otherwise.

**Response:** No citation of a specific previously published work was requested in this revision round. We therefore did not add any citation in response to such a recommendation.

**Journal Requirement 2:** Please review your reference list to ensure that it is complete and correct. If you have cited papers that have been retracted, please include the rationale for doing so in the manuscript text, or remove these references and replace them with relevant current references. Any changes to the reference list should be mentioned in the rebuttal letter that accompanies your revised manuscript.

**Response:** We reviewed the reference list for completeness and consistency. No reference was added, removed, or otherwise changed in this revision, and the reference numbering remains unchanged.

**Response to the Academic Editor Comments**

**Academic Editor Comment 1:** First, please ensure consistency in the reported threshold for the item-level content validity index (I-CVI). The Methods state that an I-CVI of ≥0.78 is considered acceptable, whereas the Results state that the observed I-CVI values of 0.833–1.000 exceeded a threshold of 0.74. Please reconcile this discrepancy and use the prespecified threshold consistently throughout the manuscript and Supporting Information.

**Response:** Thank you for identifying this inconsistency. We retained the prespecified I-CVI threshold of ≥ 0.78 reported in the Methods and corrected the Results from 0.74 to 0.78. We also clarified that this was the prespecified threshold. We reviewed the Supporting Information and found no narrative statement of an I-CVI threshold requiring revision.

**Location of change:** Results—Content validity (CVI) (page 16). The corresponding Methods statement remains on page 7.

**Revised manuscript text:** *The I-CVI ranged from 0.833 to 1.000, thus exceeding the prespecified threshold of 0.78.*

**Academic Editor Comment 2:** Second, please harmonize the interpretation of the intraclass correlation coefficient (ICC) across the Methods and Results. The Methods provide one set of interpretive thresholds for test–retest reliability, whereas the Results additionally state that an ICC >0.75 was considered acceptable and subsequently interpret values exceeding 0.70 as indicating good reliability. Please use a single prespecified interpretive framework consistently throughout the manuscript and avoid introducing a different threshold in the Results unless it was defined a priori and appropriately referenced.

**Response:** Thank you for highlighting this inconsistency. We retained the prespecified ICC interpretive framework reported in the Methods (0.70–1.00 indicating excellent stability) and removed the additional statement that an ICC > 0.75 was considered acceptable from the Results. We revised the Results to interpret the total and domain ICCs consistently as excellent stability and clarified that the two-way mixed model used agreement. The Abstract was also changed from “good” to “excellent” to align with the same framework.

**Locations of changes:** Abstract—Results (page 1); Results—Reliability analysis (page 18). The prespecified interpretive framework remains in Methods—Statistical analysis (page 10).

**Revised manuscript text:** *The overall scale showed good internal consistency reliability (Cronbach’s α = 0.893, split-half reliability = 0.885), and test–retest reliability among the 39 participants included in the analysis was excellent (intraclass correlation coefficient = 0.965).*

**Revised manuscript text:** *Test–retest reliability was evaluated in the 39 participants included in the analysis using ICCs with a two-way mixed model with agreement. The ICC for the total score of the scale was 0.965 (95% CI: 0.933–0.982), with the ICCs for each dimension (95% CI) being 0.937 (0.880–0.967), 0.963 (0.959–0.989), and 0.970 (0.942–0.985), all within the prespecified range of 0.70–1.00, indicating excellent stability.*

**Academic Editor Comment 3:** Please also conduct one final consistency check of the revised manuscript and Supporting Information to ensure that terminology, statistical thresholds, and interpretations are fully aligned and that the ethics statement entered in the submission system is identical to that reported in the manuscript.

**Response:** We completed a final consistency review of the clean manuscript, the manuscript with tracked changes, and the Supporting Information. Terminology, statistical thresholds, and interpretations relevant to the present revision are aligned. The clean manuscript contains no tracked changes or editorial comments, and accepting all revisions in the marked manuscript yields text identical to the clean manuscript. The Supporting Information contains no narrative I-CVI or ICC threshold statements requiring amendment. We will ensure at resubmission that the ethics statement entered in the submission system is identical to the statement in the Methods.

**Location checked:** Throughout the manuscript and Supporting Information; Methods—Ethical considerations (page 10); submission-system ethics field at resubmission.

**Ethics statement to be entered in the submission system:** *This study was approved by the Ethics Committee of the First Hospital of Changzhou City, Jiangsu Province (ethics approval number: 2024-099). Written informed consent was obtained from all participants. The researchers informed participants of the study purpose and procedures before data collection. Participation was voluntary, and participants were assured that they could withdraw from the study without providing a reason. Data were kept confidential and used solely for research purposes.*

We again thank the Academic Editor for the careful review and constructive guidance.
