# Supplementary material for: Development and preliminary psychometric evaluation of the hemodialysis patient care dependency scale (HD-CDS)
Source: PLoS One. 2026 Sep 15;21(9):e0358118. doi: 10.1371/journal.pone.0358118 (PMC13577502; doi:10.1371/journal.pone.0358118)
Supplement: Peer review history

# Peer review: Decision Letter 0

**DOI:** 10.1371/journal.pone.0358118.r001

**Authors:** Diana Laila Ramatillah

7 May 2026

Dear Dr. Liang

Thank you for submitting your manuscript to PLOS ONE. After careful consideration, we feel that it has merit but does not fully meet PLOS ONE’s publication criteria as it currently stands. Therefore, we invite you to submit a revised version of the manuscript that addresses the points raised during the review process.

Please submit your revised manuscript by Jun 21 2026 11:59PM. If you will need more time than this to complete your revisions, please reply to this message or contact the journal office at plosone@plos.org. When you're ready to submit your revision, log on to https://www.editorialmanager.com/pone/ and select the 'Submissions Needing Revision' folder to locate your manuscript file.

- A letter that responds to each point raised by the academic editor and reviewer(s). You should upload this letter as a separate file labeled 'Response to Reviewers'.
- A marked-up copy of your manuscript that highlights changes made to the original version. You should upload this as a separate file labeled 'Revised Manuscript with Track Changes'.
- An unmarked version of your revised paper without tracked changes. You should upload this as a separate file labeled 'Manuscript'.

If you would like to make changes to your financial disclosure, please include your updated statement in your cover letter. Guidelines for resubmitting your figure files are available below the reviewer comments at the end of this letter.

If applicable, we recommend that you deposit your laboratory protocols in protocols.io to enhance the reproducibility of your results. Protocols.io assigns your protocol its own identifier (DOI) so that it can be cited independently in the future. For instructions see: https://journals.plos.org/plosone/s/submission-guidelines#loc-laboratory-protocols. Additionally, PLOS ONE offers an option for publishing peer-reviewed Lab Protocol articles, which describe protocols hosted on protocols.io. Read more information on sharing protocols at https://plos.org/protocols?utm_medium=editorial-email&utm_source=authorletters&utm_campaign=protocols.

As the corresponding author, your ORCID iD is verified in the submission system and will appear in the published article. PLOS supports the use of ORCID, and we encourage all coauthors to register for an ORCID iD and use it as well. Please encourage your coauthors to verify their ORCID iD within the submission system before final acceptance, as unverified ORCID iDs will not appear in the published article. *Only*  the individual author can complete the verification step; PLOS staff *cannot*  verify ORCID iDs on behalf of authors.

We look forward to receiving your revised manuscript.

Kind regards,

Diana Laila Ramatillah, PhD

Academic Editor

PLOS One

Journal Requirements:

When submitting your revision, we need you to address these additional requirements.

1. Please ensure that your manuscript meets PLOS ONE's style requirements, including those for file naming. The PLOS ONE style templates can be found at

https://journals.plos.org/plosone/s/file?id=wjVg/PLOSOne_formatting_sample_main_body.pdf and

https://journals.plos.org/plosone/s/file?id=ba62/PLOSOne_formatting_sample_title_authors_affiliations.pdf.

2. Thank you for stating the following financial disclosure:

“Science and Technology Project for the Development and Enhancement of Nursing Discipline at The First People's Hospital of Changzhou, yy2023001 and yy2023015.”

Please state what role the funders took in the study.  If the funders had no role, please state: "The funders had no role in study design, data collection and analysis, decision to publish, or preparation of the manuscript."

If this statement is not correct you must amend it as needed.

Please include this amended Role of Funder statement in your cover letter; we will change the online submission form on your behalf.

3. We note that your Data Availability Statement is currently as follows: [All relevant data are within the manuscript and its Supporting Information files. ]

Please confirm at this time whether or not your submission contains all raw data required to replicate the results of your study. Authors must share the “minimal data set” for their submission. PLOS defines the minimal data set to consist of the data required to replicate all study findings reported in the article, as well as related metadata and methods (https://journals.plos.org/plosone/s/data-availability#loc-minimal-data-set-definition).

For example, authors should submit the following data:

- The values behind the means, standard deviations and other measures reported;

- The values used to build graphs;

- The points extracted from images for analysis.

Authors do not need to submit their entire data set if only a portion of the data was used in the reported study.

If your submission does not contain these data, please either upload them as Supporting Information files or deposit them to a stable, public repository and provide us with the relevant URLs, DOIs, or accession numbers. For a list of recommended repositories, please see https://journals.plos.org/plosone/s/recommended-repositories.

If there are ethical or legal restrictions on sharing a de-identified data set, please explain them in detail (e.g., data contain potentially sensitive information, data are owned by a third-party organization, etc.) and who has imposed them (e.g., an ethics committee). Please also provide contact information for a data access committee, ethics committee, or other institutional body to which data requests may be sent. If data are owned by a third party, please indicate how others may request data access.

4. Please be informed that funding information should not appear in the Acknowledgments section or other areas of your manuscript. We will only publish funding information present in the Funding Statement section of the online submission form. Please remove any funding-related text from the manuscript.

5. Your ethics statement should only appear in the Methods section of your manuscript. If your ethics statement is written in any section besides the Methods, please delete it from any other section.

6. If the reviewer comments include a recommendation to cite specific previously published works, please review and evaluate these publications to determine whether they are relevant and should be cited. There is no requirement to cite these works unless the editor has indicated otherwise.

Additional Editor Comments (if provided):

1. Please mention in the method how many participants in this research

2. Please make analysis between sociodemographic and the scoring

3. Explain in the discussion about correlation sociodemographic and the scoring

[Note: HTML markup is below. Please do not edit.]

Reviewers' comments:

Reviewer's Responses to Questions

**Comments to the Author**

1. Is the manuscript technically sound, and do the data support the conclusions?

Reviewer #1: Yes

Reviewer #2: Yes

Reviewer #3: Yes

Reviewer #4: Yes

**********

2. Has the statistical analysis been performed appropriately and rigorously?

Reviewer #1: Yes

Reviewer #2: Yes

Reviewer #3: Yes

Reviewer #4: Yes

**********

3. Have the authors made all data underlying the findings in their manuscript fully available?

The PLOS Data policy

Reviewer #1: No

Reviewer #2: Yes

Reviewer #3: No

Reviewer #4: No

**********

4. Is the manuscript presented in an intelligible fashion and written in standard English?

Reviewer #1: Yes

Reviewer #2: Yes

Reviewer #3: Yes

Reviewer #4: No

**********

Reviewer #1: The manuscript demonstrates high technical rigor, appropriate statistical analysis, and strong psychometric evidence. Addressing the data availability issue and clarifying study dates will make it fully compliant with PLOS ONE criteria. Regarding more details, I have added an attachment file including what i must be check and the reasoning for all responses above.

Reviewer #2: I have carefully reviewed the manuscript and find it to be clear and well‑structured The arguments are coherent, the methodology is appropriate, and the conclusions are consistent with the presented analysis.

Reviewer #3: I am not sure if the authors have provided all the data that partain to this study because a link was not provided to be able to assess the data. They only said that it can be provided on reasonable request. The manuscript discussed topical issue relating to the care of patients with chronic kidney disease. Correcting the issues mentioned in the review comment will enrich the rigosity of the manuscript.

Reviewer #4: It is a great work with great public health significance. It is a work with relevant clinical problem, clear innovation, large validation sample, multiple development steps, and strong reliability statistics with acceptable factor analysis.

However, below are major points that should be considered

1. The manuscript needs serious professional editing. Examples: “may be crucial” looks too weak; “was also accepted” feels awkward; “we conceptualize” has tense issue; “assessment work better guarantees” has incorrect grammar. Additionally, there are many spacing/punctuation issues

= I recommend the authors to use native English editing.

2. In submission form it says all relevant data are within manuscript and supporting files. But in manuscript it says “Data available from corresponding author on reasonable request”. These contradict each other.

= The authors should fix this.

3. There are some issues with timeline consistency. The Methods says “Study conducted August 2024 to December 2024”. But recruitment says 01/12/2022 to 01/10/2024.

= Need correction or explanation.

4. The criterion validity needs better Justification. You used Barthel Index with correlation = -0.715. It is reasonable, but Barthel measures ADL, not dialysis dependency. Why you used Barthel instead of nursing workload or dependency gold standard?

= This needs stronger explanation.

5. Some Statistical Descriptions Are Weak. Examples: “ICC was accepted”, and “good discriminative ability”.

= It needs confidence intervals for alpha if possible. And also need clearer rationale for retaining Item 3.3 despite low correlation.

And below are minor comments

6. Ethical Statement Needs Minor Editing. Use: Written informed consent was obtained from all participants. Not “consents”.

7. All patients are from one hospital in Changzhou. This limits generalizability. So, it needs stronger limitation statement.

8. Who exactly completed HD-CDS? Sometimes says nurses scored it. Sometimes patients participated. This needs clarity. Is it observer-rated? Nurse-administered? Patient interview?

9. The current title too long. Better: Development and Psychometric Validation of the Hemodialysis Patient Care Dependency Scale (HD-CDS)

**********

what does this mean?). If published, this will include your full peer review and any attached files.

If you choose “no”, your identity will remain anonymous but your review may still be made public.

**Do you want your identity to be public for this peer review?**  For information about this choice, including consent withdrawal, please see our Privacy Policy

Reviewer #1: **Yes:** Abdelrahim Dafalla Elhag Ahmed

Reviewer #2: No

Reviewer #3: No

Reviewer #4: No

**********

[NOTE: If reviewer comments were submitted as an attachment file, they will be attached to this email and accessible via the submission site. Please log into your account, locate the manuscript record, and check for the action link "View Attachments". If this link does not appear, there are no attachment files.]

To ensure your figures meet our technical requirements, please review our figure guidelines: https://journals.plos.org/plosone/s/figures

You may also use PLOS’s free figure tool, NAAS, to help you prepare publication quality figures: https://journals.plos.org/plosone/s/figures#loc-tools-for-figure-preparation.

NAAS will assess whether your figures meet our technical requirements by comparing each figure against our figure specifications.

Attachment Submitted filename: Reviewing for Manuscript Number PONE_D_25_67462.docx

Attachment Submitted filename: Review Report PONE-D-25-67462.docx

# Author comment: Author response to Decision Letter 1

**DOI:** 10.1371/journal.pone.0358118.r002

1 Jun 2026

Response to Journal Requirements：

Journal Requirement 1: Please ensure that your manuscript meets PLOS ONE's style requirements, including those for file naming. The PLOS ONE style templates can be found at https://journals.plos.org/plosone/s/file?id=wjVg/PLOSOne_formatting_sample_main_body.pdf and https://journals.plos.org/plosone/s/file?id=ba62/PLOSOne_formatting_sample_title_authors_affiliations.pdf.

Response: Thank you for this reminder. We have carefully checked and revised the manuscript according to the PLOS ONE style requirements and formatting templates, including the title page, main text structure, references, tables/figures, and file naming. In addition, we requested professional English editing and journal-specific formatting support from LetPub to ensure that the revised manuscript conforms to the requirements of PLOS ONE. The revised clean manuscript and the marked-up manuscript have been prepared and named according to the journal’s submission requirements.

Journal Requirement 2: Thank you for stating the following financial disclosure: “Science and Technology Project for the Development and Enhancement of Nursing Discipline at The First People's Hospital of Changzhou, yy2023001 and yy2023015.”

Please state what role the funders took in the study. If the funders had no role, please state: “The funders had no role in study design, data collection and analysis, decision to publish, or preparation of the manuscript.” If this statement is not correct you must amend it as needed.

Please include this amended Role of Funder statement in your cover letter; we will change the online submission form on your behalf.

Response: Thank you for your reminder. We have included the amended Role of Funder statement in the cover letter as requested. The statement is as follows: “This study was supported by the Science and Technology Project for the Development and Enhancement of Nursing Discipline at The First People's Hospital of Changzhou, yy2023001 and yy2023015. The funders had no role in study design, data collection and analysis, decision to publish, or preparation of the manuscript.” In accordance with the journal’s requirements, funding-related text has been removed from the manuscript and will only be provided in the cover letter and the Funding Statement section of the online submission form.

Response: Thank you for your reminder. We have included the amended Role of Funder statement in the cover letter as requested. The statement is as follows: “This study was supported by the Science and Technology Project for the Development and Enhancement of Nursing Discipline at The First People's Hospital of Changzhou, yy2023001 and yy2023015. The funders had no role in study design, data collection and analysis, decision to publish, or preparation of the manuscript.” In accordance with the journal’s requirements, funding-related text has been removed from the manuscript and will only be provided in the cover letter and the Funding Statement section of the online submission form.

Journal Requirement 3: We note that your Data Availability Statement is currently as follows: “All relevant data are within the manuscript and its Supporting Information files.” Please confirm at this time whether or not your submission contains all raw data required to replicate the results of your study. Authors must share the “minimal data set” for their submission.

Response: Thank you for your reminder. We confirm that our submission contains all anonymized raw data required to replicate the findings reported in this study. The minimal data set has been uploaded as Supporting Information files, including S1–S4 Excel files. Specifically, S1_Data contains participant demographic and clinical characteristics, item scores for item analysis and reliability/validity analyses, and criterion validity (Barthel Index) data; S2_Data contains the raw ratings for content validity (I-CVI); S3_Data contains the test-retest scores for ICC analysis; and S4_Data contains the detailed statistical output for the analyses summarized in Table 5. We have revised the Data Availability Statement accordingly. The revised statement can be found in the Revised Manuscript with Tracked Changes and Professional English Editing, Page 23, Lines 21–27.

Journal Requirement 4: Please be informed that funding information should not appear in the Acknowledgments section or other areas of your manuscript. We will only publish funding information present in the Funding Statement section of the online submission form. Please remove any funding-related text from the manuscript.

Response: Thank you for your clarification. We have removed all funding-related text from the manuscript, including the Acknowledgments section and any other areas of the manuscript. The funding information and the Role of Funder statement have been provided in the cover letter as requested and will be included in the Funding Statement section of the online submission form.The funding statement originally located on Page 23 has been removed.

Journal Requirement 5: Your ethics statement should only appear in the Methods section of your manuscript. If your ethics statement is written in any section besides the Methods, please delete it from any other section.

Response: Thank you for your reminder. We have carefully checked the manuscript and ensured that the ethics statement appears only in the Methods section. The retained ethics statement is presented under the subsection “Ethical considerations” in the Revised Manuscript with Tracked Changes, Page 9, Lines 25–31, and Page 10, Lines 1–2. The duplicate ethics statement previously included outside the Methods section at the end of the manuscript has been removed. The deletion can be found in the Revised Manuscript with Tracked Changes, Page 23.

Journal Requirement 6: If the reviewer comments include a recommendation to cite specific previously published works, please review and evaluate these publications to determine whether they are relevant and should be cited. There is no requirement to cite these works unless the editor has indicated otherwise.

Response: Thank you for this clarification. We have carefully reviewed the reviewer comments and confirm that no reviewer requested or recommended the citation of specific previously published works. Therefore, no additional references were added in response to this requirement.

Additional Editor Comment 1: Please mention in the method how many participants in this research.

Response: Thank you for your suggestion. We have clarified the number of participants in the revised manuscript. The number of participants is mentioned in the Abstract, Page 1, Line 15, and further described in the Methods section, Page 8, Line 12. Specifically, the revised manuscript states that 395 participants were included in the psychometric evaluation, and that the retest sample accounted for 10% of the total sample (n = 40).

Additional Editor Comment 2: Please make analysis between sociodemographic and the scoring.

Response: Thank you for your suggestion. We have added analyses examining the associations between sociodemographic/clinical variables and HD-CDS total scores. The statistical methods used for these analyses have been described in the Statistical analysis section of the Revised Manuscript with Tracked Changes, Page 9, Lines 19–24. The results are presented in the Results section under “Correlates of HD-CDS scores” and in Table 5, both of which can be found in the Revised Manuscript with Tracked Changes, Page 18.

Additional Editor Comment 3: Explain in the discussion about correlation sociodemographic and the scoring.

Response: Thank you for your suggestion. We have added an explanation in the Discussion section regarding the relationships between sociodemographic/clinical variables and HD-CDS scores. We discussed that patients with lower education levels, more frequent HD sessions, and temporary or more complex vascular access types tended to have higher care dependency scores. We also explained the negative correlations of dialysis vintage and pre-dialysis blood pressure with care dependency, and noted that sex, age, and ultrafiltration rate were not significantly associated with HD-CDS scores. This explanation can be found in the Revised Manuscript with Tracked Changes, Page 19, Line 26 to Page 20, Line 9.

Response to Reviewer Comments

We sincerely thank the reviewer for the positive evaluation of our manuscript and for recognizing the value of this study. We are grateful for the reviewer’s constructive comments and suggestions, which have helped us further improve the clarity, rigor, and overall quality of the manuscript. We have carefully addressed each comment and revised the manuscript accordingly. Detailed point-by-point responses are provided below.

Reviewer Comment 1: The manuscript needs serious professional editing. Examples: “may be crucial” looks too weak; “was also accepted” feels awkward; “we conceptualize” has tense issue; “assessment work better guarantees” has incorrect grammar. Additionally, there are many spacing/punctuation issues. I recommend the authors to use native English editing.

Response: Thank you for your valuable comment. We fully agree that the language quality of the manuscript needed substantial improvement. After completing all revisions and additions in response to the editor’s and reviewers’ comments, we submitted the revised manuscript to LetPub for professional English language editing by native English-speaking editors. The manuscript has been thoroughly edited for grammar, wording, tense consistency, punctuation, spacing, and overall readability. The examples mentioned by the reviewer, as well as similar language issues throughout the manuscript, have been corrected. We have also provided the language editing certificate from LetPub as supporting documentation.

Reviewer Comment 2: In submission form it says all relevant data are within manuscript and supporting files. But in manuscript it says “Data available from corresponding author on reasonable request”. These contradict each other. The authors should fix this.

Response: Thank you for pointing out this inconsistency. We have revised the Data Availability Statement to ensure consistency between the manuscript and the submission form. The previous statement indicating that data were available from the corresponding author upon reasonable request has been removed. The revised manuscript now states that all anonymized raw data required to replicate the findings of this study have been uploaded as Supporting Information files, including S1–S4 Excel files. This correction has also been described in detail in our response to Journal Requirement 3.

Reviewer Comment 3: There are some issues with timeline consistency. The Methods says “Study conducted August 2024 to December 2024”. But recruitment says 01/12/2022 to 01/10/2024. Need correction or explanation.

Response: Thank you for pointing out this inconsistency. We apologize for this error. The dates reported in the Participants section were incorrect. The correct timeline of the study is as follows: the scale development phase was conducted from August 1, 2024, to September 30, 2024, and the psychometric evaluation phase was conducted from October 1, 2024, to December 1, 2024.

We have revised the Design section to clearly state the two stages and their specific dates: “This study was conducted from August 2024 to December 2024 using an exploratory sequential mixed methods design. The research comprised two stages: (1) scale development (August 1, 2024, to September 30, 2024) and (2) psychometric evaluation (October 1, 2024, to December 1, 2024), which included refining the scale and assessing its psychometric properties.”

We have also corrected the Participants section. The original incorrect statement, “from 01/12/2022 to 01/10/2024,” has been replaced with: “Patient recruitment and data collection spanned from October 1, 2024, to December 1, 2024.” All timeline inconsistencies have therefore been corrected in the revised manuscript. These revisions can be found in the Revised Manuscript with Tracked Changes, Page 3, Lines 24–28.

Reviewer Comment 4: The criterion validity needs better justification. You used Barthel Index with correlation = -0.715. It is reasonable, but Barthel measures ADL, not dialysis dependency. Why you used Barthel instead of nursing workload or dependency gold standard? This needs stronger explanation.

Response: Thank you for this important comment. We agree that the rationale for using the Barthel Index as the criterion measure needed to be explained more clearly. We have added a new paragraph in the Discussion section to justify this choice. In the revised manuscript, we explain that the Barthel Index is widely used and validated for assessing activities of daily living and has conceptual overlap with care dependency, as patients with lower self-care ability generally require greater nursing assistance. We also acknowledge its limitations, including that it was not specifically designed to measure care dependency and does not capture dialysis-specific nursing needs. However, in the absence of an established gold standard for assessing care dependency among patients undergoing hemodialysis, we considered the Barthel Index to be a reasonable proxy criterion for evaluating criterion-related validity. This explanation can be found in the Revised Manuscript with Tracked Changes, Page 19, Lines 16–25.

Reviewer Comment 5: Some Statistical Descriptions Are Weak. Examples: “ICC was accepted”, and “good discriminative ability”. It needs confidence intervals for alpha if possible. And also need clearer rationale for retaining Item 3.3 despite low correlation.

Response: Thank you for these valuable suggestions. We have strengthened the statistical descriptions in the revised manuscript.

First, we added 95% confidence intervals for Cronbach’s α for the overall scale and each dimension. The revised manuscript now reports that the Cronbach’s α coefficient for the overall scale was 0.893 (95% CI: 0.877–0.908), and the coefficients for the three dimensions were 0.921 (95% CI: 0.908–0.932), 0.878 (95% CI: 0.859–0.895), and 0.737 (95% CI: 0.689–0.779), respectively. We also provided confidence intervals for split-half reliability.

Second, we revised the description of test–retest reliability and replaced the previous vague wording with a clearer criterion. In the Reliability analysis section, we now state that an ICC value greater than 0.75 was considered acceptable for test–retest reliability. The obtained ICC of 0.965 exceeded this threshold, supporting the excellent stability of the scale.

Third, we added a detailed explanation for retaining Item 3.3 despite its relatively low corrected item-total correlation. Item 3.3 showed a corrected item-total correlation of 0.313. However, it was retained for two reasons. First, qualitative findings from the nominal group technique and expert panel discussions indicated that this item was clinically important, because insufficient material or financial support from the patient’s family may increase care dependency and require nurses to compensate for unmet support needs. Second, the item’s score range was only 0–1 point, which limited its influence on the overall scale variance, and its correlation with the total score remained statistically significant (P < 0.01). Therefore, we retained Item 3.3 based on both clinical relevance and statistical significance.

The revisions regarding confidence intervals and reliability descriptions can be found in the Revised Manuscript with Tracked Changes, Page 17, Line 11 to Page 18, Line 9. The rationale for retaining Item 3.3 can be found in the Revised Manuscript with Tracked Changes, Page 14, Line 22 to Page 15, Line 3.

Reviewer Comment 6: Ethical Statement Needs Minor Editing. Use: “Written informed consent was obtained from all participants.” Not “consents”.

Response: Thank you for pointing this out. As requested in Journal Requirement 5, the ethics statement should appear only in the Methods section. Therefore, the duplicate ethics statement at the end of the manuscript, where this wording issue originally appeared, has been removed. The retained ethics statement now appears only in the Methods section under

Attachment Submitted filename: Response to Reviewers.docx

# Peer review: Decision Letter 1

**DOI:** 10.1371/journal.pone.0358118.r003

**Authors:** Wisit Kaewput

3 Aug 2026

Dear Dr. liang,

Thank you for submitting your manuscript to PLOS ONE. After careful consideration, we feel that it has merit but does not fully meet PLOS ONE’s publication criteria as it currently stands. Therefore, we invite you to submit a revised version of the manuscript that addresses the points raised during the review process.

Please submit your revised manuscript by Sep 17 2026 11:59PM. If you will need more time than this to complete your revisions, please reply to this message or contact the journal office at plosone@plos.org. When you're ready to submit your revision, log on to https://www.editorialmanager.com/pone/ and select the 'Submissions Needing Revision' folder to locate your manuscript file.

- A letter that responds to each point raised by the academic editor and reviewer(s). You should upload this letter as a separate file labeled 'Response to Reviewers'.
- A marked-up copy of your manuscript that highlights changes made to the original version. You should upload this as a separate file labeled 'Revised Manuscript with Track Changes'.
- An unmarked version of your revised paper without tracked changes. You should upload this as a separate file labeled 'Manuscript'.

If you would like to make changes to your financial disclosure, please include your updated statement in your cover letter. Guidelines for resubmitting your figure files are available below the reviewer comments at the end of this letter.

If applicable, we recommend that you deposit your laboratory protocols in protocols.io to enhance the reproducibility of your results. Protocols.io assigns your protocol its own identifier (DOI) so that it can be cited independently in the future. For instructions see: https://journals.plos.org/plosone/s/submission-guidelines#loc-laboratory-protocols. Additionally, PLOS ONE offers an option for publishing peer-reviewed Lab Protocol articles, which describe protocols hosted on protocols.io. Read more information on sharing protocols at https://plos.org/protocols?utm_medium=editorial-email&utm_source=authorletters&utm_campaign=protocols.

As the corresponding author, your ORCID iD is verified in the submission system and will appear in the published article. PLOS supports the use of ORCID, and we encourage all coauthors to register for an ORCID iD and use it as well. Please encourage your coauthors to verify their ORCID iD within the submission system before final acceptance, as unverified ORCID iDs will not appear in the published article. *Only*  the individual author can complete the verification step; PLOS staff *cannot*  verify ORCID iDs on behalf of authors.

We look forward to receiving your revised manuscript.

Kind regards,

Wisit Kaewput, MD, FRCP, FRCPSG, FASN, FAcadMEd

Academic Editor

PLOS One

Journal Requirements:

If the reviewer comments include a recommendation to cite specific previously published works, please review and evaluate these publications to determine whether they are relevant and should be cited. There is no requirement to cite these works unless the editor has indicated otherwise.

Please review your reference list to ensure that it is complete and correct. If you have cited papers that have been retracted, please include the rationale for doing so in the manuscript text, or remove these references and replace them with relevant current references. Any changes to the reference list should be mentioned in the rebuttal letter that accompanies your revised manuscript. If you need to cite a retracted article, indicate the article’s retracted status in the References list and also include a citation and full reference for the retraction notice.

Additional Editor Comments:

Thank you for the detailed point-by-point responses and the substantial revisions made to the manuscript. The manuscript is considerably clearer, and most of my previous concerns have been satisfactorily addressed. Before the manuscript can be considered further, I recommend the following minor corrections.

1. Please verify and report the test–retest sample size consistently throughout the manuscript, abstract, response letter, tables, and Supporting Information. Different sections appear to report either 39 or 40 participants. If 40 participants were invited but only 39 were included in the analysis, please state this explicitly and briefly explain the exclusion or missing assessment.

2. The ethics statement in the submission information still states, “Written informed consents were obtained from all participants.” Please revise this to, “Written informed consent was obtained from all participants.” Please also ensure that the ethics statement entered in the submission system is identical to the statement in the Methods section.

3. Because the Barthel Index is not a gold-standard measure of hemodialysis-specific care dependency, the observed correlation may be more accurately described as evidence of convergent validity rather than definitive criterion validity. Please either replace “criterion validity” with “convergent validity” throughout the Abstract, Methods, Results, Discussion, tables, and Supporting Information, or consistently describe the Barthel Index as a proxy criterion and clearly acknowledge the limitations of this terminology.

4. Some statements remain stronger than the available evidence supports, including descriptions of the HD-CDS as a “reliable and effective” or fully “validated” instrument and suggestions that it can already guide targeted nursing interventions or staffing allocation. The present study provides promising initial evidence of content validity, internal consistency, test–retest reliability, and exploratory structural validity, but it does not directly evaluate staffing decisions, nursing workload, safety outcomes, or clinical effectiveness.

Please consider revising the conclusion as follows: “The HD-CDS demonstrated promising preliminary psychometric properties in a single-center sample and may provide a useful framework for assessing care dependency among patients undergoing hemodialysis. Further multicenter studies, including independent structural validation and inter-rater reliability assessment, are required before routine clinical implementation.”

Similarly, please remove or qualify claims that the instrument has “superior” assessment capabilities or can reduce falls, infections, or other safety events unless these outcomes were directly evaluated.

5. Please identify the specific factor-extraction method used rather than reporting only the rotation method. In addition, the manuscript states that the loading threshold of 0.40 was calculated using 5.152/√(n−2). For n = 395, this calculation gives approximately 0.26 rather than 0.40. Please correct this statement. If 0.40 was selected as an a priori practical threshold, please state this directly and remove the inconsistent calculation.

6. The social-support domain had a Cronbach’s α of 0.737 and a split-half coefficient of 0.755, whereas the Methods specify values greater than 0.80 as acceptable. Please reconcile this inconsistency. The social-support domain may be described as showing moderate or acceptable preliminary internal consistency, but the manuscript should not state that every dimension exceeded the prespecified threshold. The overall scale reliability remains satisfactory.

7. Please conduct a final review of the manuscript to ensure that very small p values are reported as “p < 0.001” rather than “p = 0.000,” the domain names are used consistently throughout, duplicated or tracked-change text is removed, the clean manuscript contains no editorial comments, and the distinction between initial psychometric evaluation and external clinical validation is maintained consistently.

[Note: HTML markup is below. Please do not edit.]

Reviewers' comments:

Reviewer's Responses to Questions

**Comments to the Author**

Reviewer #2: All comments have been addressed

Reviewer #3: All comments have been addressed

Reviewer #4: All comments have been addressed

**********

2. Is the manuscript technically sound, and do the data support the conclusions?

Reviewer #2: Yes

Reviewer #3: Yes

Reviewer #4: Yes

**********

3. Has the statistical analysis been performed appropriately and rigorously?

Reviewer #2: Yes

Reviewer #3: Yes

Reviewer #4: Yes

**********

4. Have the authors made all data underlying the findings in their manuscript fully available?

The PLOS Data policy

Reviewer #2: Yes

Reviewer #3: Yes

Reviewer #4: Yes

**********

5. Is the manuscript presented in an intelligible fashion and written in standard English?

Reviewer #2: Yes

Reviewer #3: Yes

Reviewer #4: Yes

**********

Reviewer #2: The authors have addressed the earlier concerns; however, the limitation section would benefit from further elaboration, particularly regarding the justification of generalizability. The current formulation acknowledges this issue only “to some extent,” which leaves the reader without a clear understanding of how the findings can be applied beyond the study context. Strengthening this part with a more explicit and well‑reasoned justification—one that clearly aligns with and supports your final argument—is necessary before the manuscript is ready for publication.

Reviewer #3: Thank you for addressing the reviewers' comments and for the substantial revisions made to the manuscript. The revised version is considerably improved in terms of clarity, methodological reporting, and conceptual framework.

You have adequately addressed my major concerns by:

Strengthening the conceptualization of care dependency and clearly distinguishing it from frailty and functional status, while linking the concept to the three domains of the HD-CDS.

Correcting the inconsistencies in the study timeline.

Correcting the description of the item-level content validity index (I-CVI) calculation and clarifying that the analyses were performed using the standard approach.

Revising the interpretation of Kendall's W values to avoid overstating expert consensus.

I also note that the data availability statement has been revised, and the authors have indicated that the anonymized datasets supporting the findings have been provided as supporting information, in keeping with the journal's requirements.

Overall, I believe the manuscript has been substantially improved and is suitable for publication

Reviewer #4: The authors have comprehensively addressed all reviewer feedback, and the manuscript has been significantly improved. I am pleased with the final version and strongly recommend acceptance for publication.

**********

what does this mean?). If published, this will include your full peer review and any attached files.

If you choose “no”, your identity will remain anonymous but your review may still be made public.

**Do you want your identity to be public for this peer review?**  For information about this choice, including consent withdrawal, please see our Privacy Policy

Reviewer #2: No

Reviewer #3: **Yes:** Abdulakeem Ahmed

Reviewer #4: No

**********

[NOTE: If reviewer comments were submitted as an attachment file, they will be attached to this email and accessible via the submission site. Please log into your account, locate the manuscript record, and check for the action link "View Attachments". If this link does not appear, there are no attachment files.]

To ensure your figures meet our technical requirements, please review our figure guidelines: https://journals.plos.org/plosone/s/figures

You may also use PLOS’s free figure tool, NAAS, to help you prepare publication quality figures: https://journals.plos.org/plosone/s/figures#loc-tools-for-figure-preparation.

NAAS will assess whether your figures meet our technical requirements by comparing each figure against our figure specifications.

# Author comment: Author response to Decision Letter 2

**DOI:** 10.1371/journal.pone.0358118.r004

7 Aug 2026

Response to the Academic Editor and Reviewers

Manuscript ID: PONE-D-25-67462R1

Manuscript title: Development and preliminary psychometric evaluation of the hemodialysis patient care dependency scale (HD-CDS)

Dear Dr. Kaewput and Reviewers,

Thank you for the careful evaluation of our revised manuscript and for the constructive guidance. We have addressed each point below. The comments are reproduced in full, followed by our responses and the corresponding manuscript locations.

Response to Journal Requirements

Journal Requirement 1: If the reviewer comments include a recommendation to cite specific previously published works, please review and evaluate these publications to determine whether they are relevant and should be cited. There is no requirement to cite these works unless the editor has indicated otherwise.

Response: No reviewer requested citation of a specific work in this round. We therefore did not add citations solely in response to a reviewer recommendation.

Journal Requirement 2: Please review your reference list to ensure that it is complete and correct. If you have cited papers that have been retracted, please include the rationale for doing so in the manuscript text, or remove these references and replace them with relevant current references. Any changes to the reference list should be mentioned in the rebuttal letter that accompanies your revised manuscript. If you need to cite a retracted article, indicate the article’s retracted status in the References list and also include a citation and full reference for the retraction notice.

Response: We reviewed the reference list for completeness, bibliographic consistency, and retraction status. No cited item was identified as retracted. Minor hyperlink and formatting corrections were made without adding or removing any reference; the reference numbering is unchanged.

Response to Additional Editor Comments

Additional Editor Comment 1: Please verify and report the test–retest sample size consistently throughout the manuscript, abstract, response letter, tables, and Supporting Information. Different sections appear to report either 39 or 40 participants. If 40 participants were invited but only 39 were included in the analysis, please state this explicitly and briefly explain the exclusion or missing assessment.

Response: Thank you for identifying this inconsistency. Forty participants completed the two-week retest. During data verification, we found that one participant had experienced an acute gastrointestinal bleeding event during the retest interval. This clinically important change in health status violated the stability assumption for test–retest reliability, so this participant was excluded from the reliability analysis. The final test–retest analysis therefore included 39 clinically stable participants. We now report this distinction consistently in the Abstract, Methods, Results, and S3 Data. S3 Data contains 39 analyzed participants.

Location of changes: Abstract (page 1); Methods—Participants and Data collection (pages 8–9); Methods—Statistical analysis (page 9); Results—Reliability analysis (page 18); S3 Data.

Revised manuscript text: Forty participants completed a second HD-CDS assessment two weeks after the initial assessment. During data verification, one participant was found to have experienced an acute gastrointestinal bleeding event during the retest interval. Because this represented a clinically important change in health status and violated the stability assumption underlying test–retest reliability assessment, this participant was excluded. Therefore, 39 participants were included in the test–retest analysis.

Additional Editor Comment 2: The ethics statement in the submission information still states, “Written informed consents were obtained from all participants.” Please revise this to, “Written informed consent was obtained from all participants.” Please also ensure that the ethics statement entered in the submission system is identical to the statement in the Methods section.

Response: We corrected the ethics statement in the Methods section to the requested singular construction. At resubmission, we will also enter the identical sentence in the submission system so that the online statement and manuscript match exactly.

Location of changes: Methods—Ethical considerations (page 10); submission-system ethics field at resubmission.

Revised manuscript text: Written informed consent was obtained from all participants.

Additional Editor Comment 3: Because the Barthel Index is not a gold-standard measure of hemodialysis-specific care dependency, the observed correlation may be more accurately described as evidence of convergent validity rather than definitive criterion validity. Please either replace “criterion validity” with “convergent validity” throughout the Abstract, Methods, Results, Discussion, tables, and Supporting Information, or consistently describe the Barthel Index as a proxy criterion and clearly acknowledge the limitations of this terminology.

Response: We adopted the preferred terminology of convergent validity throughout the manuscript. We now explain that the Barthel Index measures activities of daily living and is conceptually related to care dependency but is not a gold-standard measure of hemodialysis-specific care dependency. We also prespecified the expected negative direction because higher HD-CDS scores indicate greater care dependency whereas higher Barthel Index scores indicate greater functional independence.

Location of changes: Abstract (page 1); Methods—Convergent validity (pages 7–8); Results—Convergent validity (page 18); Discussion (page 20); Availability of data and materials (page 23).

Revised manuscript text: Convergent validity was examined using Pearson’s correlation. The Barthel Index[27] was selected as the comparator because it assesses functional independence in activities of daily living, a construct conceptually related to care dependency. However, it does not directly assess hemodialysis-specific care needs. A negative correlation was hypothesized because higher HD-CDS scores indicate greater care dependency, whereas higher Barthel Index scores indicate greater functional independence.

Additional Editor Comment 4: Some statements remain stronger than the available evidence supports, including descriptions of the HD-CDS as a “reliable and effective” or fully “validated” instrument and suggestions that it can already guide targeted nursing interventions or staffing allocation. The present study provides promising initial evidence of content validity, internal consistency, test–retest reliability, and exploratory structural validity, but it does not directly evaluate staffing decisions, nursing workload, safety outcomes, or clinical effectiveness. Please consider revising the conclusion as follows: “The HD-CDS demonstrated promising preliminary psychometric properties in a single-center sample and may provide a useful framework for assessing care dependency among patients undergoing hemodialysis. Further multicenter studies, including independent structural validation and inter-rater reliability assessment, are required before routine clinical implementation.” Similarly, please remove or qualify claims that the instrument has “superior” assessment capabilities or can reduce falls, infections, or other safety events unless these outcomes were directly evaluated.

Response: We agree and revised the title, Abstract, Discussion, Limitations, Conclusion, and Supporting Information description to distinguish an initial psychometric evaluation from external clinical validation. We removed or qualified claims that the HD-CDS is fully validated, reliable and effective, superior, able to direct staffing, or able to reduce falls, infections, or other safety events. The Discussion now states explicitly that nursing workload, staffing allocation, clinical effectiveness, and patient-safety outcomes were not evaluated. Potential uses in care planning and resource allocation are presented as hypotheses for future study. The Conclusion uses the editor’s recommended cautious formulation.

Location of changes: Title and Abstract (pages 1–2); Discussion (pages 19–21); Limitations (pages 21–22); Conclusion (page 22); Supplementary Information description (page 23).

Revised manuscript text: The HD-CDS may provide a structured framework for describing care dependency across basic needs, HD treatment, and social support. However, the present study did not evaluate nursing workload, staffing allocation, clinical effectiveness, falls, infections, or other patient-safety outcomes. Therefore, its potential applications in individualized care planning and resource allocation should be regarded as hypotheses for future evaluation rather than established clinical benefits.

Revised manuscript text: The HD-CDS demonstrated promising preliminary psychometric properties in a single-center sample and may provide a useful framework for assessing care dependency among patients undergoing hemodialysis. Further multicenter studies, including independent structural validation and inter-rater reliability assessment, are required before routine clinical implementation.

Additional Editor Comment 5: Please identify the specific factor-extraction method used rather than reporting only the rotation method. In addition, the manuscript states that the loading threshold of 0.40 was calculated using 5.152/√(n−2). For n = 395, this calculation gives approximately 0.26 rather than 0.40. Please correct this statement. If 0.40 was selected as an a priori practical threshold, please state this directly and remove the inconsistent calculation.

Response: We now identify principal component analysis as the extraction method and orthogonal Varimax as the rotation method in the Methods, Results, and Table 4 title. We removed the inconsistent 5.152/√(n−2) calculation and state directly that a loading of at least 0.40 was selected a priori as a practical threshold for interpreting salient loadings. The statistical software remains reported as SPSS for Mac, version 27.0.1.

Location of changes: Methods—Construct validity (page 7); Methods—Statistical analysis (page 9); Results—Exploratory factor analysis and Table 4 (pages 16–17).

Revised manuscript text: Principal component analysis was used for factor extraction, followed by orthogonal Varimax rotation. … A factor loading of ≥ 0.40 was selected a priori as a practical threshold for interpreting salient loadings[25].

Additional Editor Comment 6: The social-support domain had a Cronbach’s α of 0.737 and a split-half coefficient of 0.755, whereas the Methods specify values greater than 0.80 as acceptable. Please reconcile this inconsistency. The social-support domain may be described as showing moderate or acceptable preliminary internal consistency, but the manuscript should not state that every dimension exceeded the prespecified threshold. The overall scale reliability remains satisfactory.

Response: We retained the prespecified threshold and revised the interpretation so that the results are transparent. The Results now state that the overall scale and the basic-needs and HD-treatment dimensions showed satisfactory internal consistency, whereas the social-support dimension did not meet the prespecified > 0.80 threshold and showed moderate preliminary internal consistency. We also added a caution in the Discussion that the lower social-support coefficients require examination in an independent sample.

Location of changes: Results—Reliability analysis (page 18); Discussion (page 19).

Revised manuscript text: The overall scale and the basic-needs and HD-treatment dimensions showed satisfactory internal consistency. However, the social-support dimension did not meet the prespecified threshold of > 0.80, with a Cronbach’s α of 0.737 and a split-half coefficient of 0.755, indicating moderate preliminary internal consistency.

Revised manuscript text: Although the overall scale showed satisfactory internal consistency, the lower coefficients for the social-support dimension warrant further examination in an independent sample.

Additional Editor Comment 7: Please conduct a final review of the manuscript to ensure that very small p values are reported as “p < 0.001” rather than “p = 0.000,” the domain names are used consistently throughout, duplicated or tracked-change text is removed, the clean manuscript contains no editorial comments, and the distinction between initial psychometric evaluation and external clinical validation is maintained consistently.

Response: We completed a final review of the manuscript and Supporting Information. Very small p values are now reported as p < 0.001 in the text, tables, and S4 Data. The domain names are standardized as basic needs, HD treatment, and social support, including in S2 Data. The clean manuscript contains no tracked changes or editorial comments. Duplicate text was removed, and language throughout the title, Abstract, Discussion, Limitations, Conclusion, and Supporting Information consistently distinguishes promising preliminary psychometric evidence from independent structural validation, inter-rater reliability, and external clinical validation.

Location of changes: Throughout the manuscript; Tables 2–5; S2 Data and S4 Data; with particular emphasis on the title and Abstract (pages 1–2), Discussion and Limitations (pages 19–22), and Conclusion (page 22).

Response to Reviewer #2

Reviewer #2 Comment: The authors have addressed the earlier concerns; however, the limitation section would benefit from further elaboration, particularly regarding the justification of generalizability. The current formulation acknowledges this issue only “to some extent,” which leaves the reader without a clear understanding of how the findings can be applied beyond the study context. Strengthening this part with a more explicit and well-reasoned justification—one that clearly aligns with and supports your final argument—is necessary before the manuscript is ready for publication.

Response: Thank you for this important recommendation. We substantially strengthened the Limitations section. It now explains why a single-center sample limits transportability, identifies the contextual factors that may differ across settings, states explicitly that the present findings cannot yet be generalized beyond the study setting, and specifies the multicenter and independent validation evidence needed before broader application. This wording aligns with the revised cautious Conclusion.

Location of changes: Limitations (page 21); Conclusion (page 22).

Revised manuscript text: Second, all participants were recruited from a single tertiary hospital in Changzhou, China. The patient case mix, staffing patterns, nurse training, care workflows, and local clinical practices may differ from those in other hospitals, regions, and health-care systems. Therefore, the present findings should be interpreted as preliminary evidence and cannot yet be generalized beyond the study setting. Multicenter studies involving different regions and facility levels, together with independent structural validation, are required to evaluate the external validity and transportability of the HD-CDS.

Response to Reviewer #3

Reviewer #3 Comment: Thank you for addressing the reviewers' comments and for the substantial revisions made to the manuscript. The revised version is considerably improved in terms of clarity, methodological reporting, and conceptual framework. You have adequately addressed my major concerns by: Strengthening the conceptualization of care dependency and clearly distinguishing it from frailty and functional status, while linking the concept to the three domains of the HD-CDS. Correcting the inconsistencies in the study timeline. Correcting the description of the item-level content validity index (I-CVI) calculation and clarifying that the analyses were performed using the standard approach. Revising the interpretation of Kendall's W values to avoid overstating expert consensus. I also note that the data availability statement has been revised, and the authors have indicated that the anonymized datasets supporting the findings have been provided as supporting information, in keeping with the journal's requirements. Overa

Attachment Submitted filename: Response_to_Reviewers_auresp_2.docx

# Editor report: Decision Letter 2

**DOI:** 10.1371/journal.pone.0358118.r005

**Authors:** Wisit Kaewput

11 Aug 2026

PLOS One

Dear Dr. liang,

Thank you for submitting your manuscript to PLOS ONE. After careful consideration, we feel that it has merit but does not fully meet PLOS ONE’s publication criteria as it currently stands. Therefore, we invite you to submit a revised version of the manuscript that addresses the points raised during the review process.

==============================

**ACADEMIC EDITOR:**

Thank you for submitting the revised version of your manuscript, “Development and preliminary psychometric evaluation of the hemodialysis patient care dependency scale (HD-CDS).” The manuscript has been substantially improved, and the authors have responded carefully to the previous editorial and reviewer comments. In particular, the revised manuscript provides clearer reporting of the test–retest sample, appropriately characterizes the association with the Barthel Index as convergent validity, clarifies the factor extraction and rotation procedures, moderates claims regarding validation and clinical application, and provides a more appropriately cautious discussion of generalizability.

Only a small number of editorial and methodological consistency issues remain before the manuscript can be considered for acceptance.

First, please ensure consistency in the reported threshold for the item-level content validity index (I-CVI). The Methods state that an I-CVI of ≥0.78 is considered acceptable, whereas the Results state that the observed I-CVI values of 0.833–1.000 exceeded a threshold of 0.74. Please reconcile this discrepancy and use the prespecified threshold consistently throughout the manuscript and Supporting Information.

Second, please harmonize the interpretation of the intraclass correlation coefficient (ICC) across the Methods and Results. The Methods provide one set of interpretive thresholds for test–retest reliability, whereas the Results additionally state that an ICC >0.75 was considered acceptable and subsequently interpret values exceeding 0.70 as indicating good reliability. Please use a single prespecified interpretive framework consistently throughout the manuscript and avoid introducing a different threshold in the Results unless it was defined a priori and appropriately referenced.

Please also conduct one final consistency check of the revised manuscript and Supporting Information to ensure that terminology, statistical thresholds, and interpretations are fully aligned and that the ethics statement entered in the submission system is identical to that reported in the manuscript.

These are minor issues and do not require substantial additional analysis. A careful revision addressing the points above should place the manuscript in a strong position for final consideration. A point-by-point response indicating the changes made would be appreciated.

==============================

Please submit your revised manuscript by Sep 25 2026 11:59PM. If you will need more time than this to complete your revisions, please reply to this message or contact the journal office at plosone@plos.org. When you're ready to submit your revision, log on to https://www.editorialmanager.com/pone/ and select the 'Submissions Needing Revision' folder to locate your manuscript file.

- A letter that responds to each point raised by the academic editor and reviewer(s). You should upload this letter as a separate file labeled 'Response to Reviewers'.
- A marked-up copy of your manuscript that highlights changes made to the original version. You should upload this as a separate file labeled 'Revised Manuscript with Track Changes'.
- An unmarked version of your revised paper without tracked changes. You should upload this as a separate file labeled 'Manuscript'.

If you would like to make changes to your financial disclosure, please include your updated statement in your cover letter. Guidelines for resubmitting your figure files are available below the reviewer comments at the end of this letter.

If applicable, we recommend that you deposit your laboratory protocols in protocols.io to enhance the reproducibility of your results. Protocols.io assigns your protocol its own identifier (DOI) so that it can be cited independently in the future. For instructions see: https://journals.plos.org/plosone/s/submission-guidelines#loc-laboratory-protocols. Additionally, PLOS ONE offers an option for publishing peer-reviewed Lab Protocol articles, which describe protocols hosted on protocols.io. Read more information on sharing protocols at https://plos.org/protocols?utm_medium=editorial-email&utm_source=authorletters&utm_campaign=protocols.

As the corresponding author, your ORCID iD is verified in the submission system and will appear in the published article. PLOS supports the use of ORCID, and we encourage all coauthors to register for an ORCID iD and use it as well. Please encourage your coauthors to verify their ORCID iD within the submission system before final acceptance, as unverified ORCID iDs will not appear in the published article. *Only*  the individual author can complete the verification step; PLOS staff *cannot*  verify ORCID iDs on behalf of authors.

We look forward to receiving your revised manuscript.

Kind regards,

Wisit Kaewput, MD, FRCP, FRCPSG, FASN, FAcadMEd

Academic Editor

PLOS One

Journal Requirements:

If the reviewer comments include a recommendation to cite specific previously published works, please review and evaluate these publications to determine whether they are relevant and should be cited. There is no requirement to cite these works unless the editor has indicated otherwise.

Please review your reference list to ensure that it is complete and correct. If you have cited papers that have been retracted, please include the rationale for doing so in the manuscript text, or remove these references and replace them with relevant current references. Any changes to the reference list should be mentioned in the rebuttal letter that accompanies your revised manuscript. If you need to cite a retracted article, indicate the article’s retracted status in the References list and also include a citation and full reference for the retraction notice.

Additional Editor Comments:

Thank you for submitting the revised version of your manuscript, “Development and preliminary psychometric evaluation of the hemodialysis patient care dependency scale (HD-CDS).” The manuscript has been substantially improved, and the authors have responded carefully to the previous editorial and reviewer comments. In particular, the revised manuscript provides clearer reporting of the test–retest sample, appropriately characterizes the association with the Barthel Index as convergent validity, clarifies the factor extraction and rotation procedures, moderates claims regarding validation and clinical application, and provides a more appropriately cautious discussion of generalizability.

Only a small number of editorial and methodological consistency issues remain before the manuscript can be considered for acceptance.

First, please ensure consistency in the reported threshold for the item-level content validity index (I-CVI). The Methods state that an I-CVI of ≥0.78 is considered acceptable, whereas the Results state that the observed I-CVI values of 0.833–1.000 exceeded a threshold of 0.74. Please reconcile this discrepancy and use the prespecified threshold consistently throughout the manuscript and Supporting Information.

Second, please harmonize the interpretation of the intraclass correlation coefficient (ICC) across the Methods and Results. The Methods provide one set of interpretive thresholds for test–retest reliability, whereas the Results additionally state that an ICC >0.75 was considered acceptable and subsequently interpret values exceeding 0.70 as indicating good reliability. Please use a single prespecified interpretive framework consistently throughout the manuscript and avoid introducing a different threshold in the Results unless it was defined a priori and appropriately referenced.

Please also conduct one final consistency check of the revised manuscript and Supporting Information to ensure that terminology, statistical thresholds, and interpretations are fully aligned and that the ethics statement entered in the submission system is identical to that reported in the manuscript.

These are minor issues and do not require substantial additional analysis. A careful revision addressing the points above should place the manuscript in a strong position for final consideration. A point-by-point response indicating the changes made would be appreciated.

[Note: HTML markup is below. Please do not edit.]

[NOTE: If reviewer comments were submitted as an attachment file, they will be attached to this email and accessible via the submission site. Please log into your account, locate the manuscript record, and check for the action link "View Attachments". If this link does not appear, there are no attachment files.]

To ensure your figures meet our technical requirements, please review our figure guidelines: https://journals.plos.org/plosone/s/figures

You may also use PLOS’s free figure tool, NAAS, to help you prepare publication quality figures: https://journals.plos.org/plosone/s/figures#loc-tools-for-figure-preparation.

NAAS will assess whether your figures meet our technical requirements by comparing each figure against our figure specifications.

# Author comment: Author response to Decision Letter 3

**DOI:** 10.1371/journal.pone.0358118.r006

12 Aug 2026

Response to the Academic Editor

Manuscript ID: PONE-D-25-67462R2

Manuscript title: Development and preliminary psychometric evaluation of the hemodialysis patient care dependency scale (HD-CDS)

Dear Dr. Kaewput,

Thank you for the careful evaluation of our revised manuscript and for identifying the remaining editorial and methodological consistency issues. We have addressed each point below. Because the Additional Editor Comments repeat the Academic Editor comments verbatim, each distinct concern is reproduced and answered once in this response.

Response to Journal Requirements

Journal Requirement 1: If the reviewer comments include a recommendation to cite specific previously published works, please review and evaluate these publications to determine whether they are relevant and should be cited. There is no requirement to cite these works unless the editor has indicated otherwise.

Response: No citation of a specific previously published work was requested in this revision round. We therefore did not add any citation in response to such a recommendation.

Journal Requirement 2: Please review your reference list to ensure that it is complete and correct. If you have cited papers that have been retracted, please include the rationale for doing so in the manuscript text, or remove these references and replace them with relevant current references. Any changes to the reference list should be mentioned in the rebuttal letter that accompanies your revised manuscript.

Response: We reviewed the reference list for completeness and consistency. No reference was added, removed, or otherwise changed in this revision, and the reference numbering remains unchanged.

Response to the Academic Editor Comments

Academic Editor Comment 1: First, please ensure consistency in the reported threshold for the item-level content validity index (I-CVI). The Methods state that an I-CVI of ≥0.78 is considered acceptable, whereas the Results state that the observed I-CVI values of 0.833–1.000 exceeded a threshold of 0.74. Please reconcile this discrepancy and use the prespecified threshold consistently throughout the manuscript and Supporting Information.

Response: Thank you for identifying this inconsistency. We retained the prespecified I-CVI threshold of ≥ 0.78 reported in the Methods and corrected the Results from 0.74 to 0.78. We also clarified that this was the prespecified threshold. We reviewed the Supporting Information and found no narrative statement of an I-CVI threshold requiring revision.

Location of change: Results—Content validity (CVI) (page 16). The corresponding Methods statement remains on page 7.

Revised manuscript text: The I-CVI ranged from 0.833 to 1.000, thus exceeding the prespecified threshold of 0.78.

Academic Editor Comment 2: Second, please harmonize the interpretation of the intraclass correlation coefficient (ICC) across the Methods and Results. The Methods provide one set of interpretive thresholds for test–retest reliability, whereas the Results additionally state that an ICC >0.75 was considered acceptable and subsequently interpret values exceeding 0.70 as indicating good reliability. Please use a single prespecified interpretive framework consistently throughout the manuscript and avoid introducing a different threshold in the Results unless it was defined a priori and appropriately referenced.

Response: Thank you for highlighting this inconsistency. We retained the prespecified ICC interpretive framework reported in the Methods (0.70–1.00 indicating excellent stability) and removed the additional statement that an ICC > 0.75 was considered acceptable from the Results. We revised the Results to interpret the total and domain ICCs consistently as excellent stability and clarified that the two-way mixed model used agreement. The Abstract was also changed from “good” to “excellent” to align with the same framework.

Locations of changes: Abstract—Results (page 1); Results—Reliability analysis (page 18). The prespecified interpretive framework remains in Methods—Statistical analysis (page 10).

Revised manuscript text: The overall scale showed good internal consistency reliability (Cronbach’s α = 0.893, split-half reliability = 0.885), and test–retest reliability among the 39 participants included in the analysis was excellent (intraclass correlation coefficient = 0.965).

Revised manuscript text: Test–retest reliability was evaluated in the 39 participants included in the analysis using ICCs with a two-way mixed model with agreement. The ICC for the total score of the scale was 0.965 (95% CI: 0.933–0.982), with the ICCs for each dimension (95% CI) being 0.937 (0.880–0.967), 0.963 (0.959–0.989), and 0.970 (0.942–0.985), all within the prespecified range of 0.70–1.00, indicating excellent stability.

Academic Editor Comment 3: Please also conduct one final consistency check of the revised manuscript and Supporting Information to ensure that terminology, statistical thresholds, and interpretations are fully aligned and that the ethics statement entered in the submission system is identical to that reported in the manuscript.

Response: We completed a final consistency review of the clean manuscript, the manuscript with tracked changes, and the Supporting Information. Terminology, statistical thresholds, and interpretations relevant to the present revision are aligned. The clean manuscript contains no tracked changes or editorial comments, and accepting all revisions in the marked manuscript yields text identical to the clean manuscript. The Supporting Information contains no narrative I-CVI or ICC threshold statements requiring amendment. We will ensure at resubmission that the ethics statement entered in the submission system is identical to the statement in the Methods.

Location checked: Throughout the manuscript and Supporting Information; Methods—Ethical considerations (page 10); submission-system ethics field at resubmission.

Ethics statement to be entered in the submission system: This study was approved by the Ethics Committee of the First Hospital of Changzhou City, Jiangsu Province (ethics approval number: 2024-099). Written informed consent was obtained from all participants. The researchers informed participants of the study purpose and procedures before data collection. Participation was voluntary, and participants were assured that they could withdraw from the study without providing a reason. Data were kept confidential and used solely for research purposes.

We again thank the Academic Editor for the careful review and constructive guidance.

Attachment Submitted filename: Response_to_Reviewers_auresp_3.docx

# Editor report: Decision Letter 3

**DOI:** 10.1371/journal.pone.0358118.r007

**Authors:** Wisit Kaewput

27 Aug 2026

Development and preliminary psychometric evaluation of the hemodialysis patient care dependency scale (HD-CDS)

PONE-D-25-67462R3

Dear Dr. liang,

We’re pleased to inform you that your manuscript has been judged scientifically suitable for publication and will be formally accepted for publication once it meets all outstanding technical requirements.

Within one week, you’ll receive an e-mail detailing the required amendments. When these have been addressed, you’ll receive a formal acceptance letter and your manuscript will be scheduled for publication.

An invoice will be generated when your article is formally accepted. Please note, if your institution has a publishing partnership with PLOS and your article meets the relevant criteria, all or part of your publication costs will be covered. Please make sure your user information is up-to-date by logging into Editorial Manager at Editorial Manager® and clicking the ‘Update My Information' link at the top of the page. For questions related to billing, please contact billing support.

If your institution or institutions have a press office, please notify them about your upcoming paper to help maximize its impact. If they’ll be preparing press materials, please inform our press team as soon as possible -- no later than 48 hours after receiving the formal acceptance. Your manuscript will remain under strict press embargo until 2 pm Eastern Time on the date of publication. For more information, please contact onepress@plos.org.

Kind regards,

Wisit Kaewput, MD, FRCP, FRCPSG, FASN, FAcadMEd

Academic Editor

PLOS One

Additional Editor Comments (optional):

Accept as is.

Reviewers' comments:

# Editor report: Acceptance letter

**DOI:** 10.1371/journal.pone.0358118.r008

**Authors:** Wisit Kaewput

PONE-D-25-67462R3

PLOS One

Dear Dr. liang,

I'm pleased to inform you that your manuscript has been deemed suitable for publication in PLOS One. Congratulations! Your manuscript is now being handed over to our production team.

At this stage, our production department will prepare your paper for publication. This includes ensuring the following:

* All references, tables, and figures are properly cited

* All relevant supporting information is included in the manuscript submission,

* There are no issues that prevent the paper from being properly typeset

You will receive further instructions from the production team, including instructions on how to review your proof when it is ready. Please keep in mind that we are working through a large volume of accepted articles, so please give us a few days to review your paper and let you know the next and final steps.

Lastly, if your institution or institutions have a press office, please let them know about your upcoming paper now to help maximize its impact. If they'll be preparing press materials, please inform our press team within the next 48 hours. Your manuscript will remain under strict press embargo until 2 pm Eastern Time on the date of publication. For more information, please contact onepress@plos.org.

You will receive an invoice from PLOS for your publication fee after your manuscript has reached the completed accept phase. If you receive an email requesting payment before acceptance or for any other service, this may be a phishing scheme. Learn how to identify phishing emails and protect your accounts at https://explore.plos.org/phishing.

If we can help with anything else, please email us at customercare@plos.org.

Thank you for submitting your work to PLOS One and supporting open access.

Kind regards,

PLOS One Editorial Office Staff

on behalf of

Dr. Wisit Kaewput

Academic Editor

PLOS One
